# Transcriptome and weighted gene co-enrichment analysis revealed modules and candidate genes associated with barley response to low potassium stress

**DOI:** 10.3389/fpls.2026.1779943

**Published:** 2026-03-17

**Authors:** Kexian Zhang, Bingjie Chen, Jian Ma, Qing Lai, Deyi Hu, Yuanfeng Huo, Zhaoyong Zeng, Yinggang Xu, Yuanjie Song, Jian Zeng, Zhongwei Zhang, Haihua Xiao, Shu Yuan, Guangdeng Chen

**Affiliations:** 1 International Science and Technology Cooperation Base for Efficient Utilization of Nutrient Resources and Fertilizer Innovation, College of Resources, Sichuan Agricultural University, Chengdu, China; 2 Key Laboratory of Plant Nutrition and Agri-environment in Northwest China, Ministry of Agriculture/College of Natural Resources and Environment, Northwest A&F University, Yangling, Shaanxi, China; 3 Triticeae Research Institute, Sichuan Agricultural University, Chengdu, China

**Keywords:** barley, hub genes, low-potassium stress, transcriptome, WGCNA

## Abstract

Potassium deficiency is one of the key factors affecting crop yields. This study investigated the effects of low potassium stress on the growth of three barley varieties from physiological and biochemical indicators, transcriptomics and weighted gene co-enrichment analysis. Results indicate that low potassium treatment reduced potassium accumulation, plant height, root surface area, dry weight, and photosynthetic parameters in all barley varieties, thereby inhibiting barley growth. Significantly enhanced potassium transport coefficients in stems, along with increased H^+^,K^+^-ATPase activities, indicate that this enzyme plays a crucial role in alleviating potassium deficiency stress in barley. Transcriptome analysis indicates that low potassium treatment primarily affects hormone signal synthesis and transduction, antioxidant enzymes, and transcription factors. Differentially expressed genes are mainly involved in plant defense and immunity, metabolite and energy regulation, photosynthesis, carbohydrate and nitrogen metabolism, as well as hormone and developmental regulation. Through WGCNA analysis, 12 pivotal genes exhibiting strong interactions were identified in root-MEbrown, shoot-MEpink, and stem-MEturquoise. Five genes (*LOC123407914*, *LOC123448799*, *tplb0006k10*, *NIASHv2043B04*, *NIASHv3101N17*) belong to the same KEGG pathway: ko03040 (Splicosome), classified under the primary pathway category of Cellular Processes. These 12 genes maintain apical meristem activity and H^+^-K^+^-ATPase activity, regulate photosynthetic efficiency, maintain leaf width, ensure energy synthesis and function at the RNA helicase and nucleolar levels within the nucleus to ensure normal plant growth under low-potassium stress. Moreover, three of these genes may undergo alternative splicing events, and the effects of potassium deficiency on alternative splicing have been rarely reported. Further research on these genes may fill this gap.

## 1 Introduction

Potassium (K) is one of the essential mineral nutrients for plant growth. In plants, K ion has physiological functions such as regulating enzyme activity (Lara et al., 2020), regulating osmotic pressure (Locascio et al., 2019), maintaining charge balance (Amo et al., 2021), promoting photosynthesis (Zhang et al., 2021), regulating nitrogen metabolism (Nisha and Narender, 2022), and enhancing plant resistance to stress factors such as drought, salinity, and pathogen attack (Nieves-Cordones et al., 2019). Furthermore, most K ions in soil cannot be directly absorbed and utilized by plants, and the function of K^+^ in plants cannot be replaced by other similar ions, so K supplemented by K fertilizer is usually required for plant growth and development (Anna et al., 2008).

K fertilizer can improve water retention, nutritional value, taste, disease resistance and yield of crops (Maathuis, 2009), so it has irreplaceable effects on plants (Rawashdeh et al., 2016). In addition, Potassium fertilizer can also increase corn yields in arid regions and maintain normal corn growth under drought conditions (Cao et al., 2026). However, the use of potash fertilizer in large quantities will often cause environmental pollutants, which is not conducive to the sustainable development of agriculture (Gorostiza and Saurí, 2018). Due to the imbalance of fertilizer application and other reasons, one of the main factors restricting crop growth is the lack of K (Meena et al., 2015). When potassium supply is insufficient, plants themselves trigger various adaptive responses (Sugimura et al., 2025), evolving complex signaling networks to cope with fluctuations in soil potassium levels and adapt to potassium-deficient environments (Wang and Wu, 2013). Transcriptome analysis can better help us understand these signal networks and is one of the common methods for breeding K-efficient varieties.

Sequencing transcripts is more convenient than sequencing genomes, allowing for the generation of hypotheses related to biology, the elucidation of expression patterns, the definition of cell states, identification of genes with similar expression patterns and subsequent verification (Chen and Liao, 2017). The post-genomic era has witnessed transformative advancements in functional genomics approaches (Casamassimi et al., 2017). Many K^+^ transporters and related signaling pathways that regulate these proteins, which play an important role in nutrient uptake and transport in plants, have been discovered and their functions have been also identified and verified (Wang et al., 2020). However, there are still some unanswered questions in this field, it is of great significance to further study the genetic improvement of crop nutrient use efficiency (Römheld and Kirkby, 2010).

Transcriptomics is one of the effective methods to cultivate K-efficient varieties to cope with K shortage (Rani and Sharme, 2017). There were significant differences in LK tolerance among different plant species and among different genotypes of the same plant (Rangel and Damon, 2008). Barley is the fourth largest grain crop grown in the world after wheat, corn and rice. Cultivated barley has less allelic variation and more stable inheritance, while wild barley has better adaptability to abiotic stress (Sreenivasulu et al., 2008). The combination of the two will be beneficial to the cultivation of dominant varieties with stable inheritance of target traits (Zeng et al., 2014).

In previous studies, we conducted phenotypic screening across multiple varieties and discovered significant differences in phenotypic variation between CN0126 and other varieties. Research on Franklin and Grimmet and their progeny populations has primarily focused on certain agronomic traits and the biological stress of Fusarium crown rot (Chen et al., 2014; Chen G. D. et al., 2015; Li et al., 2015; Zheng et al., 2022). Furthermore, CN0126, as one of the subpopulations derived from Baudin and CN4027, has been extensively studied for its efficiency in phosphorus and nitrogen uptake and utilization (Guo et al., 2017; Gao et al., 2018; Gao et al., 2020; Zeng et al., 2023). K, one of the macronutrients essential for plant growth, has not been studied in these varieties. With the continuous advancement of sequencing technology, transcriptome analysis enables researchers to identify more accurate genes. Therefore, to address the previous research gap, these three varieties were selected as subjects for transcriptomic analysis under LK stress. This study established two treatments: normal potassium (NK) and low potassium (LK). Phenotypic and physiological-biochemical indicators of barley under LK stress were measured. Transcriptomic analysis and weighted gene co-expression network analysis (WGCNA) were used to investigate the effects of LK stress on the growth of the three barley varieties, aiming to identify key modules and hub genes involved in barley’s response to LK stress and thus provided a reference for the breeding of LK-tolerant barley.

## 2 Materials and methods

### 2.1 Plant material and treatment

The experiment of hydroponic cultivation was carried out in the open-air greenhouse of Chengdu Campus of Sichuan Agricultural University. The seeds of cultivated barley Grimmet, Franklin and CN0126 were sterilized with 2% H_2_O_2_ for 15min, washed with distilled water for four times, and germinated in the culture dish covered with deionized water, and Then transplant seedlings with similar growth conditions into plastic pots and cultivate them hydroponically under open greenhouse conditions. We remove the seed coat from the seedling at the time of transplantation to eliminate the K supply from the seed. Use others method to prepare hydroponic nutrient solution, with continuous aeration by air pump, setting two K treatments: low K (LK, 0.01 mM KCl) and normal K (NK, 1 mM KCl), every four days to change the culture solution, if necessary, with NaOH or HCl solution pH 5.8, the experiment adopts a randomized block design, each treatment repeated three times (Zeng J. B. et al., 2015).

### 2.2 Determination of plant physiological and biochemical indexes

After 30 days of hydroponics, photosynthetic data, plant height, root length, dry weight, total K content, and H^+^, K^+^-ATPase activity were measured in barley plants. And three replicates were determined for each treatment across all genotypes. Measure the youngest fully expanded leaves on each plant LI-6400 Portable Photosynthesis System (Li-Cor Inc.) was used from 10:00 a.m. to 13:00 a.m. Root morphology was measured by root scanner. The root system was scanned digitally (Epson perfection V700 photo, Japan) and then the root analysis system software Win RHIZO Pro V2007d (Regent Instrument Inc., Canada) analyzed root length, root surface area, root volume, and average diameter. K content was determined by H_2_SO_4_-H_2_O_2_ digestion-flame photometry. The enzymatic activities of H^+^,K^+^-ATPase were quantitatively assessed using commercial assay kits (H^+^,K^+^-ATPase Detection Kit, Shanghai Enzyme-linked Biotechnology Co., Ltd., Cat# ELK0001) following standardized protocols provided by the manufacturers, with three biological replicates per treatment condition to ensure experimental reproducibility.

### 2.3 Total RNA extraction and reverse transcription, construction of cDNA library

Total RNA was extracted from the shoot and root of barley by RNAprep Pure polysaccharide polyphenol plant total RNA extraction kit (DP441)(TIANGEN BIOTECH (BEIJING)CO., LTD), and the concentration of extracted RNA was determined by NanoDrop ND-2000. RNA extraction was performed from both shoot and root tissues of Franklin, Grimmet, and CN0126, with two treatments (LK and NK) applied. Each treatment and tissue type included three replicates, resulting in a total of 36 samples. cDNA was prepared by reverse transcription according to RR092-PrimeScript™ FAST RT reagent kit with gDNA Eraser kit instructions. The cDNA libraries were sequenced on the Illumina sequencing platform by Metware Biotechnology Co., Ltd. (Wuhan, China).

### 2.4 Quality control of sequencing data, read mapping and analysis of novel genes

The fastp tool is utilized for data quality control to guarantee the precision of subsequent analyses. Elevated base quality values signify enhanced base recognition reliability and increased accuracy (Chen et al., 2018). Clean Reads are aligned with reference genome by HISAT2 (Kim et al., 2015) to obtain position information on reference genome or gene and unique sequence characteristic information of sequencing sample, and reads are assembled into transcripts by StringTie according to position information of reads on aligned genome. The extraction of new transcript information is primarily achieved by comparing spliced transcripts with genomic annotations. The extracted results are saved in GTF (Gene Transfer Format) format. For detailed information on the GTF format, please refer to https://genome.ucsc.edu/FAQ/FAQformat.html. These new gene was aligned with KEGG (Kyoto Encyclopedia of Genes and Genomes), GO (Gene Ontology), NR (Non-Redundant Protein Database), UniProt (The Universal Protein Resource) and KOG (Clusters of orthologous groups for eukaryotic complete genomes) databases by diamond (Pertea et al., 2015).

### 2.5 Screening, functional annotation, and enrichment analysis of differential genes and enrichment analysis of gene sets

DESeq2 (Buchfink et al., 2015)was used to perform differential expression analysis between sample groups to obtain differential expression gene sets between two biological conditions, and then Benjamini-Hochberg method was used to perform multiple hypothesis test correction on hypothesis test probability (P value) to obtain False Discovery Rate (FDR) (Love et al., 2014). The screening conditions for differential genes were: |log2Fold Change|>=1 and FDR < 0.05 (Varet et al., 2016). In addition, functional enrichment analysis was performed using the GO and KEGG databases for all differentially expressed genes identified from the sequencing results. After screening different genes according to the experimental purpose, the distribution of different genes in Gene Ontology was studied by enrichment analysis to clarify the embodiment of sample differences in gene function. Pathway annotation analysis of differentially expressed genes is helpful to further interpret gene functions (Zhou et al., 2017). Gene co-enrichment analysis (GSEA) does not require specifying explicit differential gene thresholds. Instead, genes are ranked according to their differential expression in two sets of samples. Statistical methods are used to test whether a predetermined set of genes is enriched at the top or bottom of the ranking table.

### 2.6 Weighted gene co-expression network analysis

The Weighted gene co-expression network analysis (WGCNA) algorithm first assumes that gene networks follow a scale-free distribution. It defines the gene co-expression correlation matrix and the adjacency function forming the gene network, then calculates the dissimilarity coefficients between different nodes. Based on these coefficients, it constructs a hierarchical clustering tree. Different branches of this clustering tree represent distinct gene modules. Genes within the same module exhibit high co-expression levels, while genes across different modules show low co-expression levels. Similarly, the samples were divided into two parts: the shoot portion and the root portion. Each part contained barley varieties representing three genotypes. The two treatments, NK and LK, each had three replicates. The varFilter function from the R genefilter package was used to filter out lowly expressed genes and genes with stable expression levels across all samples, which improved the accuracy of gene network construction. Use the pickSoftThreshold function to calculate the optimal power value (setting R^2^ to 0.85), construct a clustering tree based on gene expression correlations and partition modules. Subsequently, the partitioned modules are correlated with phenotypic associations to identify the modules most strongly associated with the phenotype, thereby enabling gene screening. All PPI information from the sequencing results was obtained based on gene connectivity. After importing the data into an Excel spreadsheet, filter the gene interactions within the corresponding modules using the VLOOKUP function. Subsequently, perform WGCNA plotting via Metware Cloud (a free online data analysis platform) (https://cloud.metware.cn). Finally, visualize the data using Cytoscape 3.10.2 to identify pivotal genes.

### 2.7 Quantitative real-time PCR analysis

Twelve genes were selected from the transcriptome analysis data, and their expression patterns were confirmed by using qRT-PCR. Use *HvActin* as the housekeeping gene for qRT-PCR normalization. Primers were designed using Primer Premier version 5.0 from NCBI database. The delta-delta Ct (2^-ΔΔCt^) method was used to calculate the relative expression levels of DEG in different samples, using gene expression at NK levels as a control (Livak and Schmittgen, 2001).

### 2.8 Data analysis

SPSS (version 19.0) software was used to perform statistical analysis on the data of each group. Statistical significance was determined using Tukey test values P < 0.05 and significant differences were indicated by various letters.

## 3 Results

### 3.1 Change trend of physiological and biochemical indexes of barley

**Figure 1:**
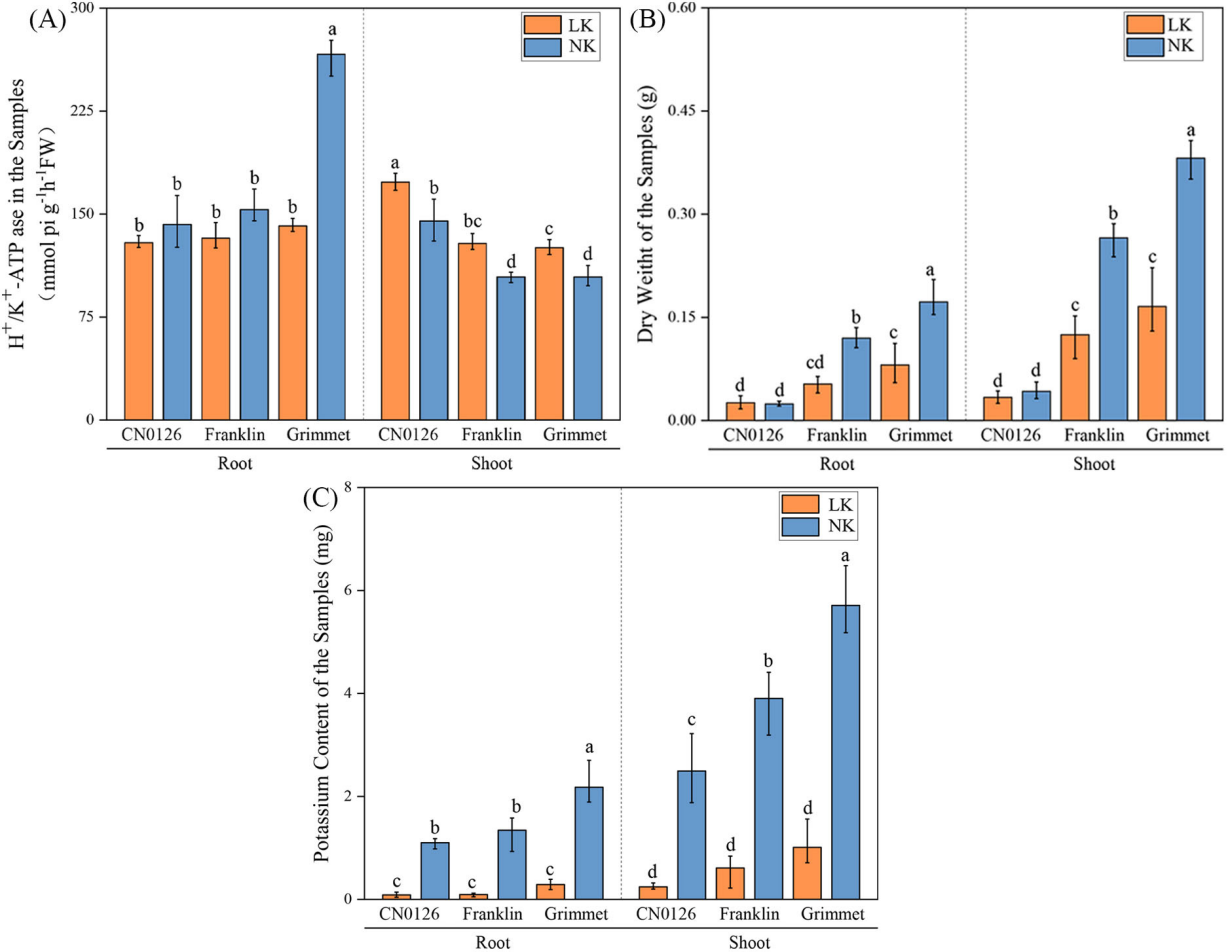
Physiological, biochemical and enzyme activity data for roots and shoot parts of three barley varieties. **(A)** H^+^,K^+^-ATPase in the root and shoot. **(B)** Dry weight of root and shoot. **(C)** Potassium content of root and shoot.

**Figure 2:**
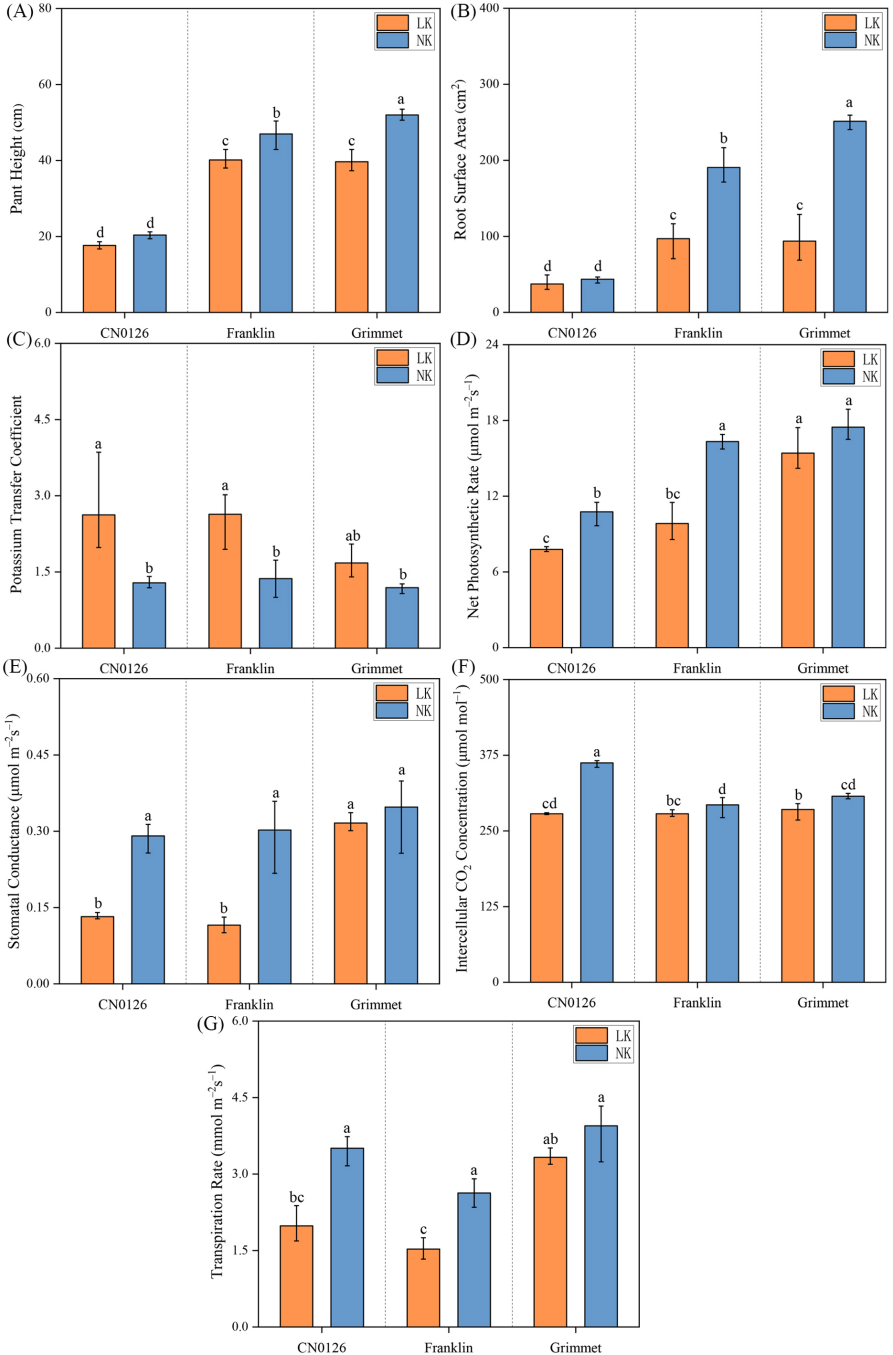
Phenotypic and photosynthetic rate index data for roots and shoots parts of three barley varieties. **(A)** Plant height. **(B)** Root surface area. **(C)** Potassium transfer coefficient. **(D)** Net photosynthetic rate. **(E)** Stomatal conductance. **(F)** Intercellular CO_2_ concentration. **(G)** Transpiration rate.

The physiological and biochemical indexes of three varieties were measured under LK and NK treatments, including dry weight, plant height and root surface area, K accumulation of shoot and root parts, and K transfer coefficent and H^+^,K^+^-ATPase activities, intercellular CO_2_ concentration, stomatal conductance, transpiration rate and net photosynthetic rate (**Figures 1**, **2**). Under LK treatment, both Franklin and Grimmet varieties exhibited significant declines in shoot and root dry weight, K accumulation, plant height, and root surface area. However, apart from K accumulation, CN0126 showed no significant changes in other trait indices such as dry weight, plant height, root surface area, and H^+^,K^+^-ATPase activities (**Figures 1**, **2**). Analysis of K transfer coefficients revealed that under LK treatment, CN0126 exhibited a significantly increased transfer coefficient. This indicates that during LK stress, the plant’s efficiency in absorbing and transporting K through its roots markedly improved, thereby mitigating the adverse effects of LK treatment on plant growth and development.

Determination of enzyme activities of these three varieties (**Figure 1A**) showed that the activities of H^+^,K^+^-ATPases increased significantly under the LK treatment. Research indicates a connection between K and plants’ light perception (Ahammed et al., 2022). Significantly, CN0126 had the highest activities of the enzymes under LK stress. Photosynthetic analysis of the three varieties showed that intercellular CO_2_ concentration of Franklin and Grimmet increased significantly under LK treatment, while CN0126 decreased significantly. However, intercellular CO_2_ concentration alone cannot explain the trend of photosynthetic changes under LK stress. Analysis of three indicators—net photosynthetic rate, stomatal conductance, and transpiration rate—revealed that CN0126 and Franklin showed significant reductions under LK treatment, while Grimet also exhibited a decrease, though not significant (**Figures 2D–G**).

### 3.2 Database quality and mapping gene analysis by RNA-seq

Paired end reads were sequenced using the Illumina platform to obtain complete transcriptome profiles of the three barley genotypes under LK and NK treatment. By sequencing cDNA library, 36 samples of three varieties produced a total of 290.79 Gb of high-quality reads, the error rate was below 0.01%, the Q30 ratio exceeded 94%, with clean reads accounting for over 85% of each sample (**Supplementary Table S1**). The clean read alignment efficiency ranged from 77.77% to 95.76% across all samples. Among samples with 40–84 million clean reads, 80.17%-91.84% mapped to unique reads, while 1.98%-6.99% mapped to multiple reads (**Supplementary Table S2**).

The RNA-seq database includes 36 samples from three barley varieties, the proportion of reads containing nitrogenous bases was below 0.01%, with adapter-related regions accounting for 5.9% to 14.25% (**Supplementary Figure S1A**; **Supplementary Table S3**). Over 83.1% of reads mapped to exons, while approximately 12% were located between introns (**Supplementary Figure S1**; **Supplementary Table S4**). All this data indicates that the base composition and distribution exhibit high quality, providing an excellent data source for subsequent analyses. The fold-proportional expression measure (FPKM), calculated as the number of fragments per million aligned reads per gene per transcript, further enhances the accuracy of gene expression quantification (**Supplementary Table S5**). The PCA plot clearly demonstrates excellent reproducibility across each treatment (**Supplementary Figure S1C**).

### 3.3 Analysis of differentially expressed genes

**Figure 3:**
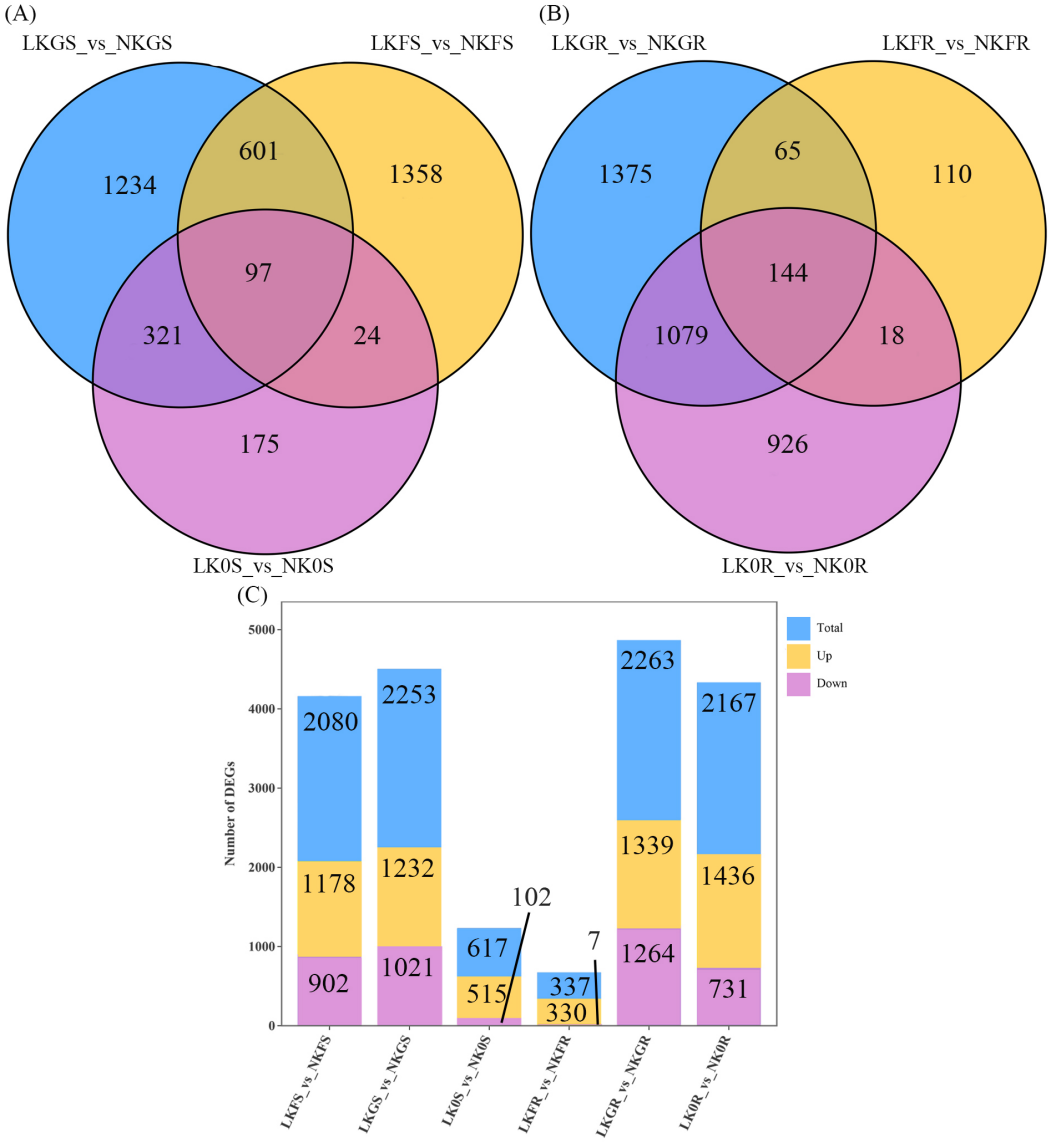
Differentially Expressed Genes (DEGs) in different grouping Venn Diagram, corresponding to the number of up-regulated and down-regulated DEGs in each group. **(A)** Venn Diagram of shoot-specific DEGs. **(B)** Venn Diagram of root-specific DEGs. **(C)** Number of Up-regulated, Down-regulated and total DEGs. Among them LKFR, LKGR, LK0R, LKFS, LKGS, LK0S represent the root and shoot parts of the Franklin, Grimmet and CN0126 varieties subject to low-potassium treatment, repectively. And NKFR, NKGR, NK0R, NKFS, NKGS, NK0S represent the root and shoot parts of the Franklin, Grimmet and CN0126 varieties under normal potassium treatment.

Sequencing results from the same region of barley plants of the same variety under LK and NK treatments were compared with the results shown in **Figure 3**. Venn plots showed that a total of 97 genes were commonly responded to in the shoot samples of the three varieties and a total of 144 genes were commonly responded to in the roots (**Figures 3A, B**). Compared to NK treatment, LK treatment resulted in the detection of 2080 differentially expressed genes (DEGs) in Franklin shoots, comprising 1178 up-regulated genes and 902 down-regulated genes. In the roots, a total of 337 DEGs were identified, with 330 genes up-regulated and 7 genes down-regulated. The Grimmet shoot sample detected a total of 2253 DEGs, comprising 1232 up-regulated genes and 1021 down-regulated genes. In the roots, 2263 DEGs were identified, including 1339 up-regulated genes and 1264 down-regulated genes. In the CN0126 shoot, a total of 617 DEGs were detected, comprising 515 up-regulated genes and 102 down-regulated genes. In the roots, 2167 DEGs were identified, including 1436 up-regulated genes and 731 down-regulated genes (**Figure 3C**).

### 3.4 Classification of GO functional annotations and KEGG pathways for DEGs

The predicted GO (Gene Ontology) annotation can further analyze the function of DEGs to identify the expression of DEGs under LK stress in barley. These genes are classified into three categories: biological process (BP), cellular component (CC) and molecular function (MF). The GO enrichment of the three barley varieties showed that the differential gene expression of the shoot and root parts was basically similar under LK stress. In the root part, biological process (BP) annotation the primary categories involved are cellular processes, metabolic processes and stimulus responses, with 2160, 1978 and 1448 DEGs, respectively. For cellular component (CC) annotation, DEGs predominantly reside in cellular anatomical entities, totaling 3044. Molecular function (MF) annotation primarily encompasses catalytic activity and binding, with 2159 and 1844 DEGs, respectively. Similar to the distribution of root GO annotations, shoot biological process (BP) annotations showed 2205, 1341 and 1139 DEGs in the three processes mentioned above, respectively. Cellular component (CC) annotation identified 3050 DEGs during the process of annotating the aforementioned cellular anatomical entities. And molecular function (MF) annotation primarily encompasses binding and catalytic activity, with 2241 and 1837 DEGs respectively (**Supplementary Figure S2**; **Supplementary Table S6**). These were mainly related to the vacuolar ion dynamic balance process, signal pathway response, the combination of catalytic activity and metabolic processes systematically reflects the response mechanism of barley to LK stress.

**Figure 4:**
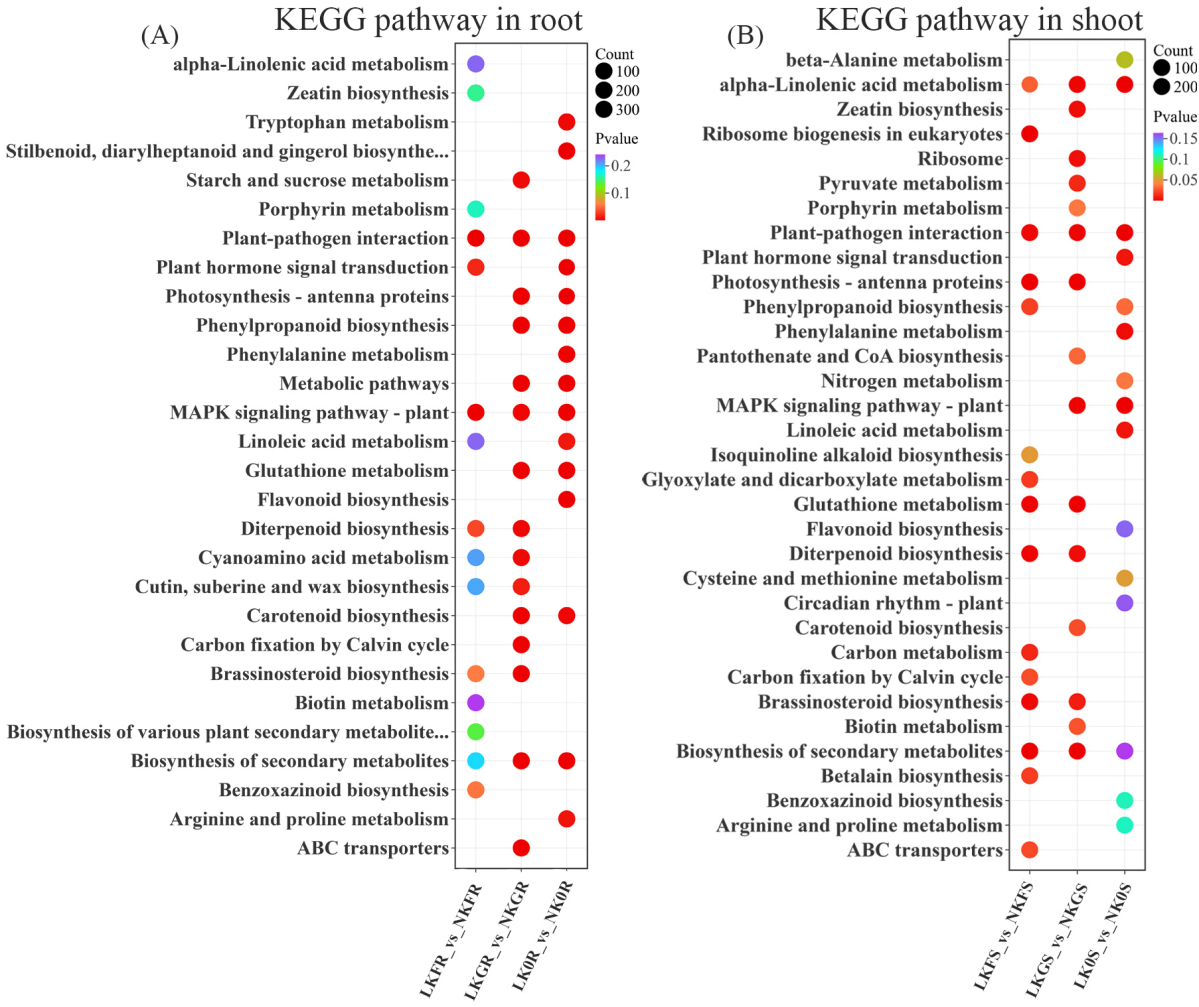
KEGG enrichment analysis plots for different sample group. **(A)** KEGG enrichment analysis of DEGs of root in Franklin, Grimmet and CN0126. **(B)** KEGG enrichment analysis of DEGs of shoot in Franklin, Grimmet and CN0126.

DEG under LK stress was also mapped to the KEGG (Kyoto Encyclopedia of Genes and Genomes) pathway database, and the top 15 pathways with the lowest Qvalue of the three varieties were summarized according to the shoot and root parts. It was found that in the shoot and root parts of these varieties showed enrichment in two KEGG pathways, which are Plant-pathogen interaction and Biosynthesis of secondary metabolites, while the roots were enriched in the MAPK signaling pathway-plant pathway in addition to the above two pathways, and the shoot parts were enriched in the alpha-linolic acid metabolism pathway (**Figure 4**; **Supplementary Table S7**). These pathways in roots respond to LK stress mainly through MAPK signaling pathways, ROS signaling pathways and CBL-CIPK signaling pathways and activate secondary metabolic synthesis genes. And pathways in shoots mitigate the effects of K deficiency on plant growth by regulating the balance of plant hormone signals, stimulating signal transduction and enhancing plant antioxidant capacity.

### 3.5 Gene enrichment analysis of fully expressed genes

Gene Set Enrichment Analysis (GSEA) does not require setting explicit thresholds for differentially expressed genes. Instead, it employs statistical methods to test whether predefined gene sets exhibit enrichment at the top or bottom of the ranking table. After enrichment analysis of gene sets and normalization of ES values, 33 GOs were found simultaneously, and their correlations were strong. Most of gene sets in aerial parts of different samples treated with the same treatment were positively associated with specific biological processes or phenotypes, but most of gene sets in aerial parts of different groups of the same sample were negatively associated with specific biological processes or phenotypes. The same ES values of shoot are not different, while biological process (BP) occupies the most among these GOs, with 17 in total, and cellular component (CC) occupies the least, with only 1, specifically expressed in protein complex process (**Supplementary Table S8**).

In the KEGG pathway enriched by GSEA, there were 132 KEGG pathways in all samples. These KEGG pathways were mainly concentrated in the metabolism of carbohydrates, lipids, amino acids, nucleotides and cofactors, as well as in the metabolism of terpenoids and alkaloids. For the 50 GSEA with the highest ES values, it was found that the aggregation in the shoot and root parts was basically similar. In the shoot part, these pathways were mainly enriched in cellular molecular metabolism, photosynthesis and cellular transportation. Unlike in the shoot part, these pathways were mainly concentrated in fatty acid metabolism, plant product biosynthesis, cellular processes and steroid and terpene biosynthesis (**Supplementary Table S9**). In the shoot and root tissues of CN0126, a protein-tagged functional gene set comprising 31 genes from GSEA-enriched GO terms exhibited the highest enrichment scores across the three varieties. Among the KEGG pathways enriched by GSEA, the Betalain biosynthesis pathway showed the highest enrichment score in the shoot parts of CN0126, encompassing 25 genes. In the roots, the Steroid biosynthesis pathway exhibited the highest enrichment score, involving 48 genes. These gene sets provide a reference for distinguishing K-sensitive and K-tolerant genotype samples.

### 3.6 Alternative splicing analysis

**Figure 5:**
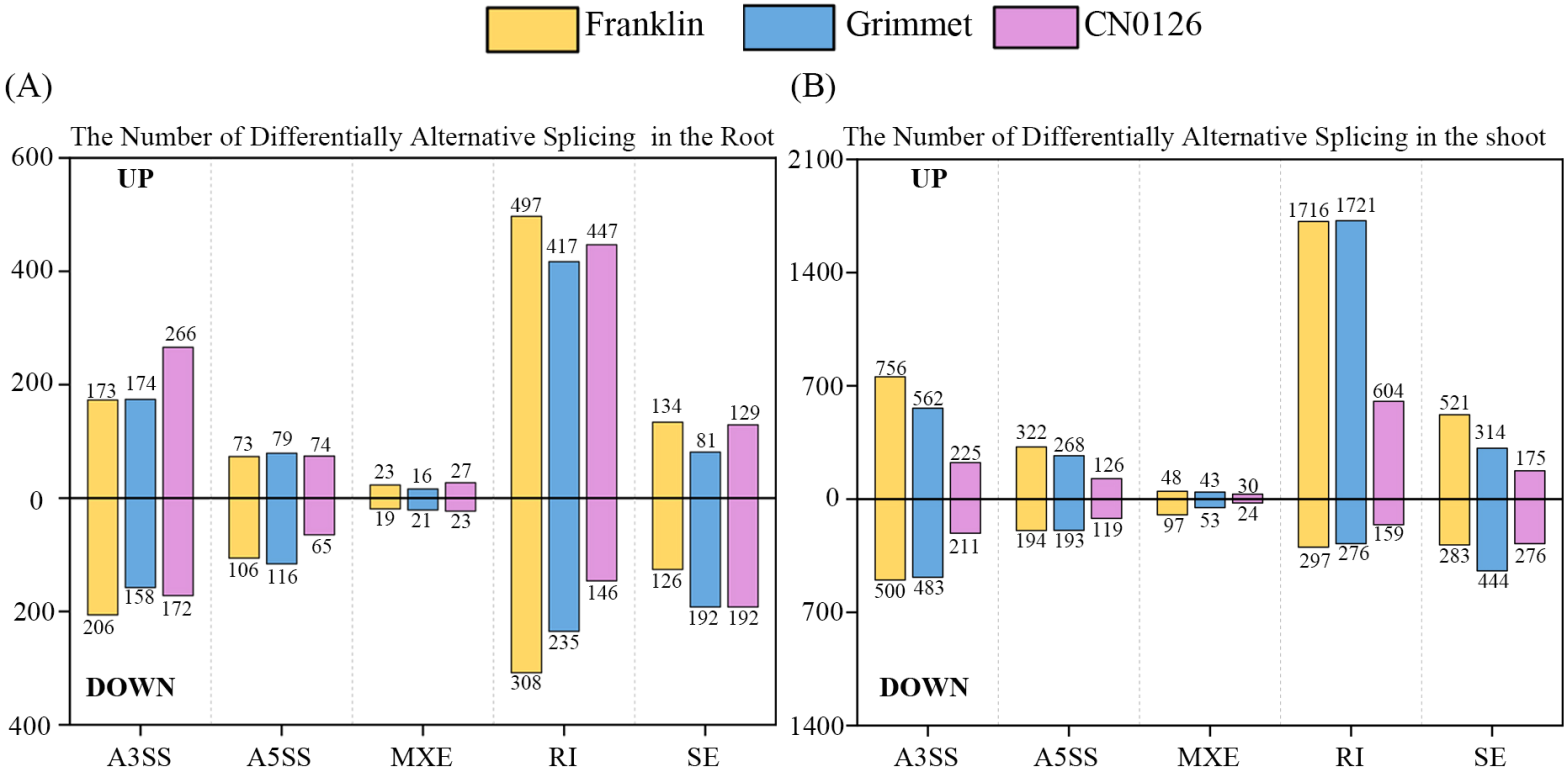
Quantify the number of differentially upregulated and downregulated Alternative Splicing (AS) events at the root and shoot levels using JCEC. **(A)** The number of differentially Alternative Splicing (AS) events quantified using JCEC at the root level. **(B)** The number of differentially Alternative Splicing (AS) events quantified using JCEC at the shoot level. Among them A3SS represents Alternative 3’ Splice Site, A5SS represents Alternative 5’ Splice Site, MXE represents Mutually Exclusive Exons, RI represents retained intron, SE represents Skipped Exon. The criterion for determining upregulation or downregulation is as follows: subtract the level of the corresponding variable shear event in the control group from the level of the variable shear event in the treatment group. If the result is greater than 0, it is considered upregulation, otherwise, it is downregulation.

Alternative Splicing (AS) is a common form of gene expression in most eukaryotic cells. An unprocessed RNA molecule can exhibit multiple forms of exon splicing, thereby increasing the complexity or adaptability of the system under physiological conditions. Therefore, We also analyzed the number of AS events associated with JCEC (Junction Count and Exon Count) across these three varieties (**Figure 5**). AS analysis of transcriptomic data was performed using rMATS (Shen et al., 2014). rMATS recognizes five main types of AS events: Skipped Exon (SE), Alternative 5’ Splice Site (A5SS), Alternative 3’ Splice Site (AS33), Mutually Exclusive Exons(MXE), Retained Intron(RI). The FRD value for differential variable shear is less than 0.05, which enhances the reliability of the results. Under LK stress, most variable shear events in both the shoot and root tissues of the three samples were up-regulated, with the number of up-regulations far exceeding that of down-regulations. The total number of AS events in the shoot portion and the number of AS events that were up-regulated were both higher than those in the root portion. The majority of these AS events were of the RI type, followed by the A3SS type. Among RI events, Franklin had the highest total number of up-regulations and down-regulations adjustments, with 497 up-regulations and 308 down-regulations, followed by Grimmet with 417 up-regulations and 235 down-regulations. CN0126 had the lowest total, with 447 up-regulations and 146 down-regulations. In shoot, Franklin also had the highest up-regulations and down-regulations count, with 1716 up-regulations and 297 down-regulations. Grimmet followed closely with 1721 up-regulations and 276 down-regulations. CN0126 had the lowest shoot count, significantly fewer than the other two, with 604 up-regulations and 159 down-regulations.

### 3.7 Weighted gene co-expression network analysis

By performing cluster analysis on genes from different samples, genes exhibiting similar expression patterns were grouped into the same module, thereby constructing a co-expression network. Similar to the preceding analysis, the three barley varieties were divided into root groups and shoot groups, and weighted gene co-enrichment analysis was performed separately for each group.

**Figure 6:**
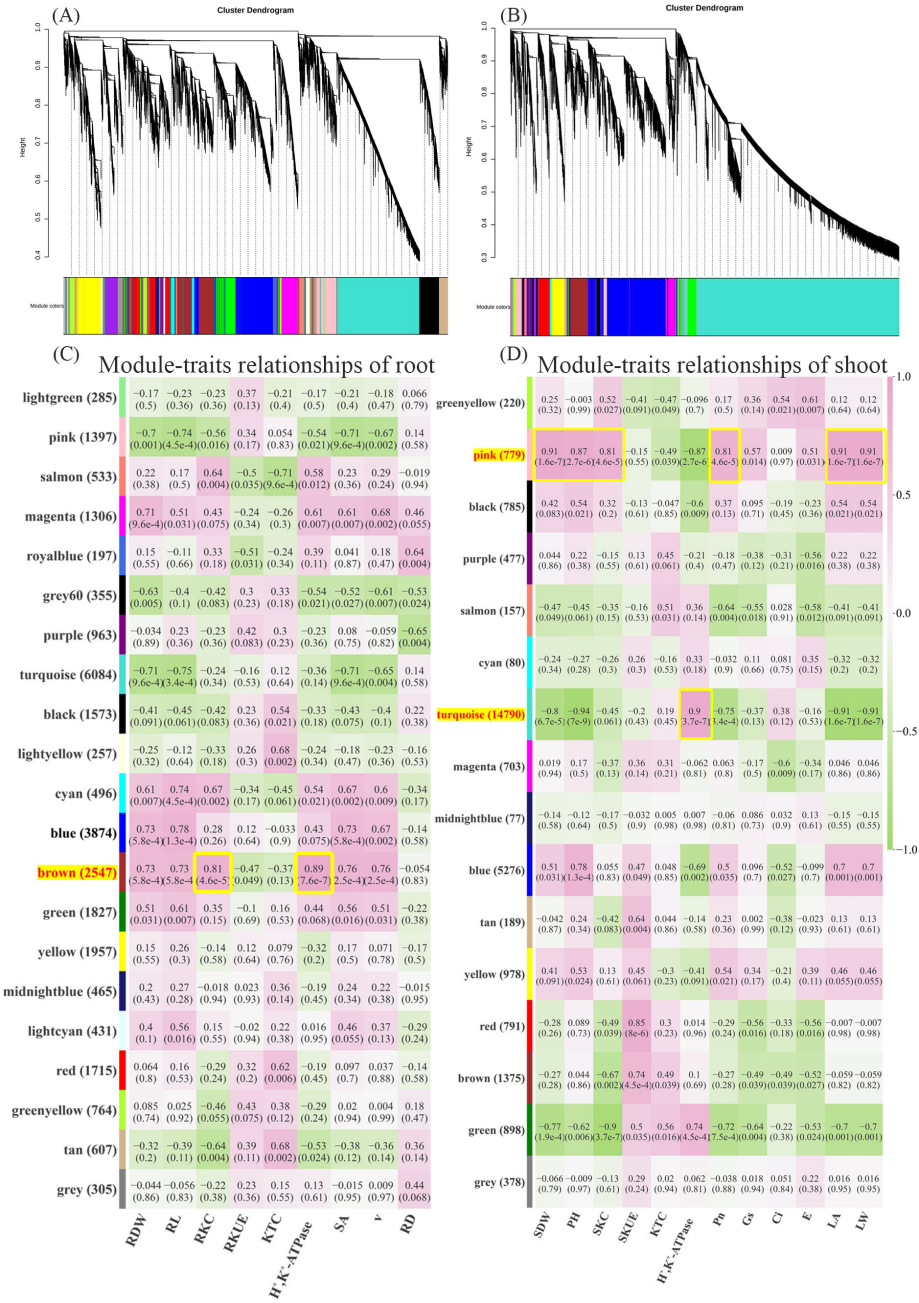
Weighted gene co-expression network analysis. **(A)** Clustering diagram of root gene expression data, where each color represents a gene module, and genes within the corresponding clustering tree belong to the same module. **(B)** Clustering diagram of shoot gene expression data. **(C)** Heatmap of correlations between different modules and root phenotypic traits and physiological indicators. **(D)** Heatmap of correlations between different modules and shoot phenotypic traits and physiological indicators. Among them R/SDW represents root/shoot dry weight, RL represents total root length, R/SKC represents potassium accumulation in roots/shoots, R/SKUE represents root/shoot potassium utilization efficiency, KTC represents potassium Transfer Coefficient, H^+^,K^+^-ATPase represents H^+^,K^+^-ATPase activity, SA represents root surface area, V represents root volume, RD represents root mean diameter; PH represents plant high, Pn represents net photosynthetic rate, Gs represents stomatal conductance, Ci represents intercellular carbon dioxide concentration, Tr represents transpiration rate, LA represents leaf surface area, LW represents Leaf width.

**Figure 7:**
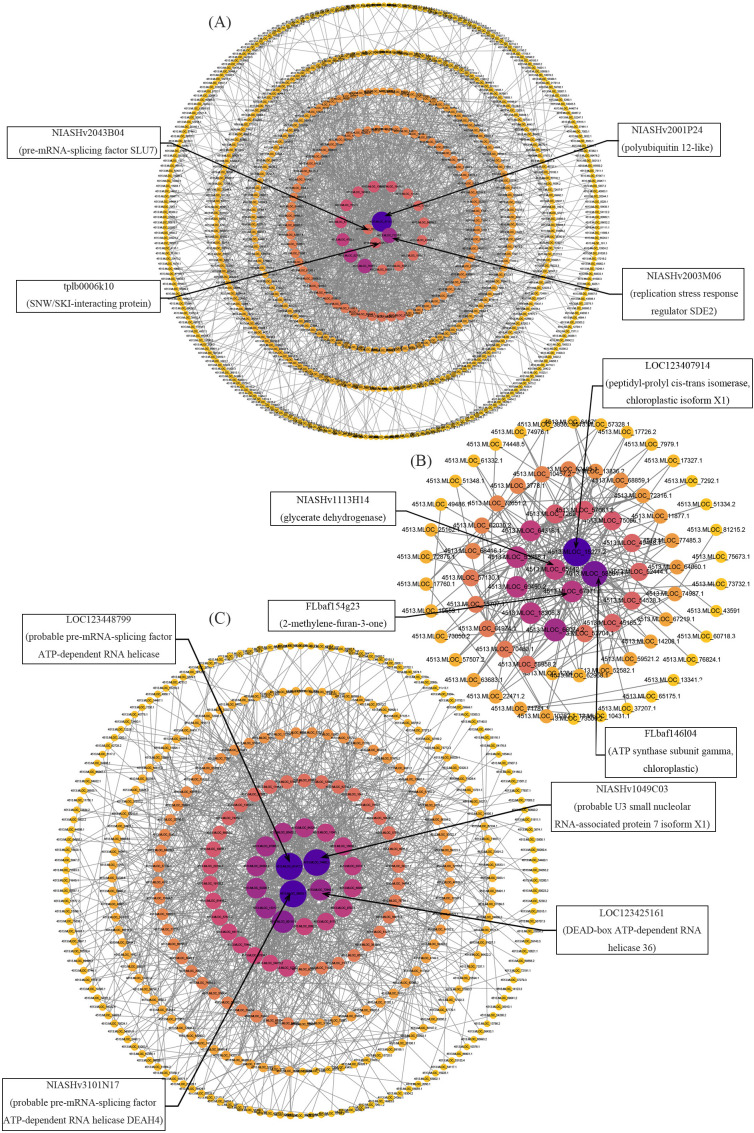
**(A)** Gene network diagram of root MEbrowen module and RKC, H^+^,K^+^-ATPase Activity-Related Genes. **(B)** Gene network diagram of the shoot MEpink module associated with SDW, PH, SKC, Pn, LA, and LW. **(C)** Gene network diagram of stem MEturquoise module associated with H^+^,K^+^-ATPase activity.

In the root, a total of 27,938 genes were grouped into 21 modules, each represented by a distinct color (**Figure 6A**). The module with the highest number of genes was MEturquoise, containing a total of 6,084 DEGs. Next was the MEbrown module, which contained 2,547 DEGs. The module with the fewest genes was MEroyalblue, containing 197 DEGs. Correlation analysis of genes within the module with different samples revealed that the MEbrown module showed the highest correlations with phenotypic traits such as RKC (r = 0.81) and H^+^,K^+^-ATPase enzyme activity (r = 0.89) (**Figure 6C**). Therefore, we performed correlation analysis between RKC data from the roots of the three varieties and the gene data in the MEbrown module for H^+^,K^+^-ATPase enzyme activity, setting a threshold of 0.85 and p ≤ 0.05, therefore it yielded the gene set within this module most strongly correlated with these two indicators (**Supplementary Table S10**). Based on these genes, we constructed a protein-protein interaction (PPI) network and found that the interactions among these genes were not significant. To further explore the relationships among genes associated with RKC and H^+^,K^+^-ATPase enzyme activities within the MEbrown module, a protein-protein interaction (PPI) analysis was conducted using all genes in the MEbrown module as templates, resulting in the construction of a PPI network. Analyze the results using Cytoscape 3.10.2, identify the top 20 hub genes via the Degree algorithm, and compare them with the results from the correlation analysis. Results indicate that among the top 20 hub genes, *NIASHv2003M06*, *tplb0006k10*, *NIASHv2043B04*, *NIASHv2001P24* showed the strongest correlations with RKC and H^+^,K^+^-ATPase activity (**Figure 7A**; **Supplementary Table S11**). These four proteins indirectly support the maintenance and efficient operation of proton pump enzyme activity by preserving root cell genomic stability, coordinating stress signaling pathways, and precisely regulating splicing networks. They also promote the active uptake and accumulation of K ions within the plant, collectively enhancing barley’s adaptability to LK environments.

In the shoot, DEGs were distributed across 16 modules totaling 27,953 genes. The module with the highest number of genes was MEturquoise, containing 14,790 DEGs, followed by the MEblue module with 5,276 DEGs. The module with the fewest genes was MEmidnightblue, comprising 77 DEGs (**Figure 6B**). Correlation analysis of genes within the module with different samples revealed that the MEpink module showed the highest correlations with phenotypic traits including SDW (r=0.91), PH (r=0.87), SKC (r=0.81), Pn (r=0.81), LA (r=0.91), and LW (r=0.91). The MEturquoise module showed the highest correlation with H^+^,K^+^-ATPase enzyme activity (r = 0.91) (**Figure 6D**). Therefore, in the shoot, we selected the MEpink and MEturquoise modules for correlation analysis with their corresponding phenotypic data. Within the MEpink module, we set a threshold of 0.85 and p ≤ 0.05 to identify the gene set most strongly correlated with the corresponding phenotype(**Supplementary Table S10**). In the MEturquoise module, due to the large number of genes, the correlation analysis set the threshold for both phenotypes to 0.90, p ≤ 0.01, therefore yielded the gene set most strongly correlated with H^+^,K^+^-ATPase enzyme activity (**Supplementary Table S10**). These genes underwent PPI analysis, visualized using Cytoscape 3.10.2, and hub genes were identified via the Degree algorithm. Within the MEpink module, the following genes were identified: *FLbaf146l04*, *NIASHv1113H14*, *FLbaf154g23* and *LOC123407914*, total of four (**Figure 7B**; **Supplementary Table S12**) hub genes with the strongest interaction relationships. Under the LK stress, these four proteins act as core components of photosynthesis and guardians of chloroplast function, collectively maintaining barley’s photosynthetic efficiency and energy metabolism homeostasis. Thus proteins play a crucial synergistic role in mitigating leaf area reduction, sustaining net photosynthetic rate, supporting plant height and aboveground dry weight growth, and optimizing K use efficiency, forming the vital physiological foundation for plants responding to hypokalemic stress.

The shoot MEturquoise module identified *NIASHv1049C03*, *LOC123425161*, *LOC123448799* and *NIASHv3101N17* totaling four hub genes (**Figure 7C**; **Supplementary Table S13**). These proteins collectively participate in regulating intracellular ion homeostasis and energy metabolism when barley encounters LK stress. H^+^,K^+^-ATPase enhances K^+^ uptake and redistribution by regulating membrane potential, thereby maintaining cellular polarity and nutrient transport. Concurrently, significant changes in the expression of numerous RNA processing, ribosomal biogenesis, and RNA helicase proteins, such as DEAD-box and DEAH family indicate that cells rapidly adapt to LK environments by regulating post-transcriptional modifications and protein synthesis. This coordinated action helps ensure energy supply and molecular stability for critical physiological processes, thereby sustaining normal barley growth under LK stress.

### 3.8 Validation of differentially expressed genes

**Figure 8:**
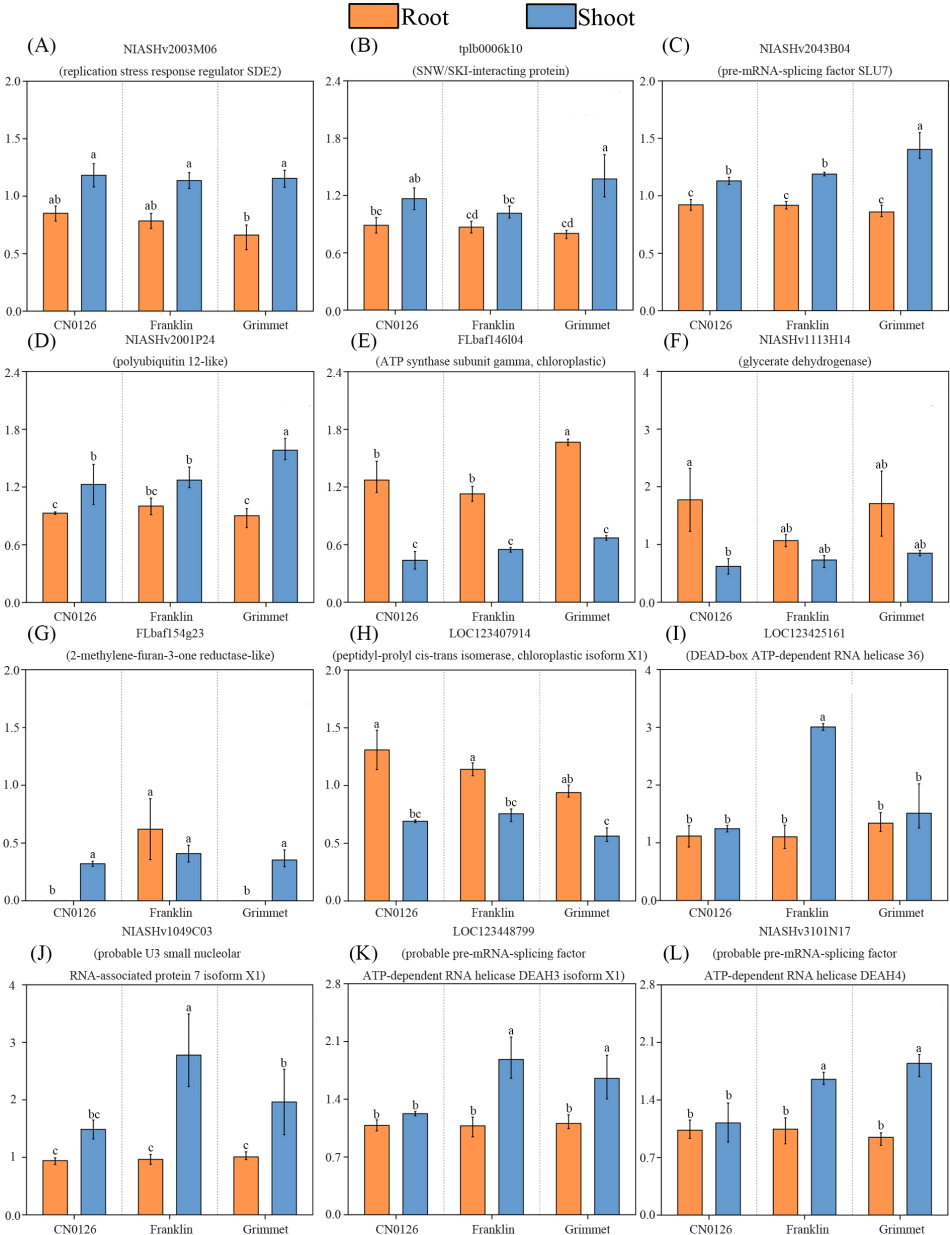
**(A–L)** Results of real-time quantitative PCR for 12 genes. The vertical axis represents the relative expression levels calculated using 2^-ΔΔCt^, where ΔCt1 denotes the difference between the target gene expression and *HvActin* expression under low-potassium treatment, and ΔCt2 denotes the difference between the target gene expression and *HvActin* expression under normal-potassium treatment.

To visually illustrate the expression trends of the aforementioned 12 genes. Using the relative gene expression levels of the NK treatment group as the control and setting its value to 1. The relative expression levels of the 12 genes are shown in **Figure 8**. These results clearly reveal the up and down-regulation patterns of these genes across the three barley varieties.

## 4 Discussion

### 4.1 Study on phenotypic and physiological-biochemical indicators of different barley

K plays a crucial role in plant growth (Imtiaz et al., 2022). Extensive research indicates that K application promotes plant growth, increasing plant height and dry weight. Conversely, K deficiency leads to a decline in both dry weight and K accumulation (Zeng et al., 2018; Ali et al., 2021; Ye et al., 2021). However, none of these studies calculated the K transfer coefficient or the activity of the relevant enzymes. In this study, Franklin, Grimmet and CN0126 barley varieties were used as experimental materials. Under LK stress, the changes of H^+^,K^+^-ATPase activities, dry weight, K content, plant height, root surface area, K transfer coefficients, and photosynthetic related data of barley were studied. Under K deficiency treatment, compared with NK treatment, both K accumulation, dry weight, plant height and root surface area of Franklin and Grimmet were significantly lower than NK treatment, and K accumulation of CN0126 also decreased significantly, but dry weight of shoot and root did not change significantly (**Figure 1**). However, CN0126 exhibited significantly enhanced K transfer coefficient and enzyme activity, with the highest content recorded. The significant improvement in these indicators may represent an effective method for plants to autonomously alleviate K deficiency symptoms.

Similarly, K also plays a crucial role in plant photosynthesis (Chen et al., 2016; Tränkner et al., 2018). In other studies, net photosynthetic rate, stomatal conductance, intercellular CO_2_ concentration, and transpiration rate generally exhibit similar trends of change when subjected to abiotic stress (Lu et al., 2016; Jákli et al., 2017; Omondi et al., 2020; Mateus et al., 2021). This is mainly because K deficiency reduces mesophyll conductivity and changes leaf anatomy, which in turn affects leaf and chloroplast surface area and reduces stomatal conductivity and chlorophyll content (Martineau et al., 2017; Singh and Reddy, 2017). Reported for other crops also proved this point through their research. Through the experiment of LK stress in corn, they confirmed that the light response pathways of PS I and II in corn seedlings were damaged under K deficiency conditions (Qu et al., 2011a), and then nitrate related enzymes, glutamate related enzymes, sucrose-phosphate synthase, phosphoenolpyruvate carboxylase, free amino acids and the synthesis activity of chlorophyll and proteins were significantly reduced (Qu et al., 2011b). In addition, Our study found that H^+^,K^+^-ATPase activities in barley shoots increased significantly when exposed to LK stress (**Figure 1**). Previous studies have shown that when K^+^ supply is insufficient, the ATP conversion efficiency of proton pumps decreases. This leads to enhanced activity of the H^+^,K^+^-ATPase to compensate for the energy deficit generated by the proton pumps (Buch-Pedersen et al., 2006). Research has found that calcium ion dependent signaling network mediated by two calcineurin plays a key role in response to K stress in plants (Tang et al., 2021), and a study on calcium ion signaling pathway in tobacco also showed that it promotes K^+^ absorption by regulating ROS homeostasis and K^+^ absorption related gene expression in plant roots under LK stress (Wang et al., 2021). Therefore, significant increases in H^+^,K^+^-ATPase activity promote K^+^ transport within plants, thereby mitigating the adverse effects of K deficiency on plant growth.

### 4.2 Analysis of differentially expressed genes among different barley varieties

Transcriptome results were analyzed for phenotypic differences in barley under LK treatment. Previous research indicates that high-quality downgraded components and longer, more precise sequences enable more accurate quantitative analysis (Hrdlickova et al., 2017; Pardo-Palacios et al., 2024). The clean reads of the three barley varieties in this study were mapped to the reference genome *Hordeum_vulgare* (https://ftp.ensemblgenomes.ebi.ac.uk/pub/plants/release-58/fasta/hordeum_vulgare/dna). Analysis of the results revealed that high-quality reads, clean reads, and unique reads accounted for a significant proportion, with extremely low error rates. The high-quality nucleotide composition demonstrated excellent reproducibility across all treatment groups. This data provides a high-quality data source and important reference for subsequent analyses.

DEG is important data that directly reflects molecular evidence of differences among samples (Liao et al., 2014). From the clustering heat map, it can be intuitively found that the differences in related gene expression between LK and NK treatment in each sample, and the overall trend of different samples with the same treatment is still consistent (**Supplementary Figure S3**). GO functional annotation and KEGG pathway analysis of DEGs are also crucial methods for screening target genes in transcriptomic analysis (Ge et al., 2008). This method has been widely applied in early transcriptomic analyses, such as in studies examining reed transcriptomics under cadmium stress (Chen et al., 2020), pearl millet transcriptomics under heat stress and drought stress (Sun et al., 2020) and peanut transcriptomics under drought stress (Jiang et al., 2021). However, these genes cannot precisely screen for specific target genes. As research progresses further, transcriptomic analysis is often combined with metabolomics or WGCNA analysis to identify pathways or hub genes with clearer functional significance (Zhang et al., 2020) (Xu et al., 2022). RNA-seq was used to analyze the phenotypic shape and genetic correlation of the shoot and root parts of three barley species to study the whole gene changes in the genome transcriptome, and we employed Weighted Gene Co-enrichment Analysis (WGCNA) to screen for hub genes expressed in barley in response to LK stress (Buchfink et al., 2015). In order to cultivate crops with improved K use efficiency to better support sustainable agriculture, it is necessary to further investigate the molecular mechanisms by which plants respond to soil K stress and improve plant K use efficiency (Li et al., 2023).

The first 20 GO annotations of Franklin in root mainly focused on phosphorelay signal transduction system, cellular responses to ethylene stimulation and ethylene-activated signaling pathway of BP. Calcium ion binding, gibberellin 2-β-dioxygenase activity and MAP kinase kinase kinase activity of CC. The first 20 GO annotations in Grimmet roots mainly focus on UDP-glycosyltransferase activity, monooxygenase activity and iron ion binding of CC. Phenylpropanoid metabolic process and secondary metabolite biosynthetic process of BP. The first 20 GO annotations in CN0126 root mainly focus on regulation of response to biotic stimulus, phenylpropanoid metabolic process and hydrogen peroxide metabolic or catabolic process of BP. Antioxidant activity, peroxidase activity and oxidoreductase activity, acting on peroxide as acceptor (**Supplementary Figure S4**). The GO annotation of DEG in roots of these three varieties is mainly distributed in several aspects of plant photosynthesis regulation, related enzyme activity and plant hormones. In addition, the functions of these genes are involved in gene expression in transcription process (Zhang X. Q. et al., 2017). The analysis of the top 20 GO annotations of DEG showed that the top 20 GO annotations of Franklin shoots mainly focused on ribonucleoprotein complex biogenesis of BP (51), nucleolus of CC (51) and monooxygenase activity of MF(50). The top 20 GO annotations for shoot of Grimmet primarily focus on three aspects of MF: hexosyltransferase activity, monooxygenase activity, and calcium ion binding, with 54, 49 and 45 annotations respectively. CN0126 in the shoot part, cellular response to alcohol, cellular response to abscisic acid stimulus, abscisic acid-activated signaling pathway and calcium ion binding were concentrated (**Supplementary Figure S5**). Moreover, each of these annotations has exactly 23 entries. Among them, the root-specific GO terms response to biotic stimuli and phenylpropanoid metabolic process, along with the shoot-specific terms cellular response to alcohol, cellular response to abscisic acid stimulus and abscisic acid-activated signaling pathway, were enriched exclusively in CN0126. These specific GO terms may play a crucial role in CN0126’s response to LK stress. The GO annotation of DEG of three varieties is enriched with calcium ion binding (GO:0005509) and oxygen response system (GO:0009768; GO:0009521;GO: 0009523;GO:0009522). When plants are exposed to LK stress, Ca^2+^ concentration increases significantly, resulting in activation of Ca^2+^-regulated protein kinase and downstream phosphorylation signal, and finally stress resistance is realized by activating stress response gene or regulating ion channel activity (Saito and Uozumi, 2020). Calcium channels located in root epidermis and root hair region can be activated by hyperpolarization of plasma membrane (PM) in response to LK stress, and then calcium sensors can be involved in K deficiency sensing (Wang X. et al., 2018). Calcium ion signaling pathway can promote the absorption and utilization efficiency of K ions in plants to cope with LK stress (Wang et al., 2021). In addition, K^+^, Ca^2+^ and ROS/RNS signaling pathways in plant growth, development and abiotic stress responses also interact, regulating ion homeostasis, antioxidant systems and stress-resistance-related gene expression in plants (Liu and Liao, 2022). A study on non-biotic stress in poplar trees revealed that GO annotations indicating significantly elevated activities of redox enzymes, peroxidases, and transporters in roots and shoots tissues, along with substantial enrichment in protein phosphorylation, cell wall modification, proteolysis, transmembrane transport, cell division and defense responses, constitute effective mechanisms for plants to cope with non-biotic stress (Georgii et al., 2019). The regulation of phenylpropanoid metabolism occurs at multiple levels, including transcriptional, post-transcriptional, post-translational and epigenetic pathways, as well as through plant hormones and molecular design strategies responding to biotic and abiotic stresses. Controlling the genetic regulation of phenylpropanoid metabolic flux has emerged as a promising approach for breeding crops with high yields, stress tolerance, and enhanced nutritional value (Dong and Lin, 2021). These studies indicate that plants adapt to environmental changes under abiotic stress through multiple signaling pathways, reactive oxygen species regulation, or modulation of various enzyme activities. This finding is largely consistent with the GO annotation enrichment patterns observed within the three barley varieties.

The results of KEGG were also the same as those of GO annotation. DEG was mainly involved in plant hormone signal transduction, plant pathogen interaction, starch and sucrose metabolism and synthesis of memory metabolites. These pathways are similarly enriched in other crops under LK stress. A study on sugarcane revealed that under LK stress, differentially expressed genes were detected in transcription factors, transporters, kinases, oxidative stress-related genes, as well as calcium ion and ethylene signaling pathways. These genes may play a key role in enhancing sugarcane’s tolerance to LK stress (Zeng Q. et al., 2015). A transcriptomic analysis of bananas under LK stress revealed that the expression of ABC transporters, protein kinases and ion transporter genes effectively alleviates K deficiency in bananas (Xu et al., 2019). Studies on K deficiency stress in tomatoes have also confirmed the crucial role these pathways play in alleviating K deficiency symptoms (Zhao et al., 2018). These studies and data show that LK stress is closely related to plant stress resistance, oxidative stress and photosynthesis. And plants activate a variety of hormone and protein regulatory mechanisms to maintain normal growth under LK stress.

Among the 33 GO annotations clustered by GSEA, Franklin has 6 GO annotations in root and 15 GO annotations in aerial part, and Grimmet has 14 GO annotations in root and 15 GO annotations in aerial part, and CN0126 has 15 GO annotations in root and 10 GO annotations in aerial part, with higher enrichment scores in aerial part of three samples (An et al., 2024). There were 10 GO annotations clustered together in the shoot of the three cultivars, and three with low false positive, which mainly played roles in photosynthesis, stress protection and metabolic rhythm of plants. The co-enrichment results of GESA showed that DEG of 14 GO annotations clustered together participated in molecular signal transduction, translation regulation and protein folding assistance in the shoot samples. There are also 17 comments that do not appear in DEG analysis, including regulatory mechanism, development and growth, signaling and communication, immunity and defense, reproduction and inheritance, host-microorganism interaction, molecular function and structure, etc. (**Supplementary Table S8**). Compared with the shoot samples, the predicted co-enrichment results in the root samples included protein translational regulation and antioxidant activity, rhythmic process, molecular signal transduction activity and aptamer activity. These DEGs are translationally regulated primarily through mRNA and trans-acting factors of cis-regulatory elements, as well as a signaling network of three conserved translational regulators TOR, SnRK1 and GCN2 (Wu et al., 2024). In addition, past research has revealed how plants use translational control to defend against viruses, regulate translation in response to metabolites, and reversibly adjust translation to various environmental parameters (Camacho et al., 2020). Research found that glutathione (GSH) can be used as a promoter to improve oxidation resistance, and proposed the mechanism of GSH regulating antioxidant system and thioglycoside metabolism pathway, which provided a basis for plant antioxidant protection research (Zhu et al., 2022). Functional analysis of DEGs indicates that they dominate genes in nutrient pool, carbohydrate metabolism and defense proteins during plant development (Kaushik et al., 2020), and differences in plant nutrient redistribution, gene expression and sink-source relationship may explain different accumulation patterns of these nutrient stores (Ren et al., 2022).

### 4.3 WGCNA analysis

WGCNA is widely used for inferring co-expression network modules and serves as a primary method for gene expression clustering (Zhang et al., 2024). It has been extensively utilized in screening key cadmium-tolerant genes in the auxin pathway of Lycium barbarum (Yang et al., 2025), screening key salt tolerance genes in the lignin and mustard oil biosynthesis pathways of sand onion (Cao et al., 2025), Screening of Key Regulatory Genes Involved in Sugarcane Photosynthesis During Drought (Wang et al., 2025) and Screening of Genes Affecting Maize Plant Height (Wang H. S. et al., 2018). In order to find the major regulatory genes in response to LK stress in three barley varieties, this study employed weighted gene co-expression network analysis (WGCNA) to analyze differentially expressed genes (DEGs). WGCNA was employed to perform gene-trait correlation analysis on the root and stem tissues of three barley varieties. The results revealed significant positive correlations between phenotypic traits reflecting plant growth status and gene clusters within different modules across different barley tissues.

After performing correlation analyses between phenotypic traits in these three modules and their corresponding genes, we constructed protein-protein interaction (PPI) networks using the selected gene sets. Analysis of the results was conducted using Cytoscape 3.10.2 and Degree algorithm was applied to identify a total of 12 hub genes across the three modules. Within the root-specific MEbrown module, four hub genes were identified: *NIASHv2003M06, tplb0006k10, NIASHv2043B04, NIASHv2001P24*. The proteins predicted for these genes in the NCBI database are respectively: Silencing-defective 2 (SDE2) (*NIASHv2003M06*) (Jo et al., 2016), SNW/SKI-interacting protein (*tplb0006k10*) (Jiang et al., 2022), pre-mRNA-splicing factor SLU7 (Splicing factor synergistic lethal with U5 snRNA 7) (*NIASHv2043B04*) (Jiménez et al., 2019) and polyubiquitin 12-like (*NIASHv2001P24*) (Niu et al., 2023). The function of *tplb0006k10* has been studied in several crops. The homologue of the SNW/SKIP co-regulator encoded by maize SAT2 cDNA confers enhanced salt tolerance to yeast. Furthermore, ectopic expression of the AtSKIP gene modulates salt tolerance, desiccation resistance, and abscisic acid sensitivity induced in *Arabidopsis* under stress conditions. Green fluorescent protein-tagged AtSKIP localizes to the nuclei of onion cells and transgenic *Arabidopsis* cells (Lim et al., 2010). Another study indicates that the expression of this gene enhances wheat’s adaptive mechanisms to phosphorus deficiency (Oono et al., 2013). However, the other three genes and their homologs have not been extensively studied, yet there remain numerous reports regarding the proteins they are predicted to encode. These four proteins were shown to exhibit the strongest correlations with RKC and H^+^,K^+^-ATPase enzyme activity. DE2 is a PCNA-associated protein that functions as a replication stress response factor. By regulating the fork protection complex (FPC), it plays a crucial role in maintaining active replication and counteracting replication stress, thereby preventing root apical meristem cells from losing their proliferative capacity due to genomic damage. This ensures sustained root growth activity and the continued expression of proton pump-related genes (Rageul et al., 2020). This effectively maintains the K^+^ uptake function of plant roots and may be the key protein responsible for sustaining K accumulation in plants. At the same time, H^+^,K^+^-ATPase enzymes establish a transmembrane proton gradient through polarized localization in the extracellular membranes of root epidermal and endodermal cells, driving K^+^ uptake mediated by high-affinity K transporters such as the HAK/KUP family (Li et al., 2022). Under LK conditions, SLU7, as key pre-mRNA splicing regulators, may modulate the generation of splicing variants for H^+^,K^+^-ATPase itself or its upstream signaling molecules such as calcium-sensing proteins and 14-3–3 proteins, thereby optimizing proton pump activation efficiency and membrane localization (Chen Q. et al., 2015). This enhances the activity of the H^+^,K^+^-ATPase enzyme, thereby increasing the plant’s ability to absorb K^+^ from the external environment. By regulating gene expression stability and alternative splicing, they synergistically maintain root function and K accumulation capacity under LK stress. Furthermore, these expressed proteins may also respond to K transport-related genes, enhancing plants’ perception and utilization of limited K resources.

In the shoot MEpink module, we identified four hub genes—*FLbaf146l04*, *FLbaf154g23*, *NIASHv1113H14* and *LOC123407914*—that showed significant correlations with SDW, PH, SKC, Pn, LA and LW. The proteins predicted for these genes in the NCBI database are respectively: ATP synthase subunit gamma chloroplastic (*FLbaf146l04*), peptidyl-prolyl cis-trans isomerase chloroplastic isoform X1 (*FLbaf154g23*), glycerate dehydrogenase (*NIASHv1113H14*), 2-methylene-furan-3-one reductase-like (*LOC123407914*). The four predicted genes encoding these proteins have not yet been reported. Adenosine triphosphate (ATP) synthases are dynamos that interconvert rotational and chemical energy. The γ-subunit in spinach contains an L-shaped double-hairpin structure, which functions as a redox sensor, preventing the wasteful hydrolysis of ATP during nighttime and reducing the plant’s energy expenditure (Hahn et al., 2018). Furthermore, the ATP synthase γ subunit directly participates in the function of photosystem I/II and ATP synthesis (Pateraki et al., 2013). Hypokalemic stress disrupts thylakoid membrane structure and inhibits electron transport efficiency (Che et al., 2022). Leaf width directly reflects the strength of a plant’s photosynthetic capacity. Previous studies have shown that recombinant expression of Prolyl cis-trans isomerase in cells enhances their tolerance to temperature and salt stress, indicating that this protein plays a crucial role in stress tolerance mechanisms (Subin et al., 2016). Prolyl cis-trans isomerase participates in the correct folding of proteins, assists in the assembly of photosynthetic complexes within chloroplasts, and maintains normal photosynthetic function in plants under LK stress. Glycerate dehydrogenase is involved in carbon metabolism pathways. Research has proposed a hypothesis that among the two serine biosynthetic pathways in plants, one regulates redox balance in stressed plant cells by participating in reactions related to glycerate and phosphorylated serine pathways. These pathways serve as crucial processes linking carbon and nitrogen metabolism while maintaining cellular redox and energy levels under stress conditions (Igamberdiev and Kleczkowski, 2018). 2-Methylenefuran-3-one reductase is an enzyme that catalyzes the reduction of 2-methylenefuran-3-one, participating in the biosynthesis of aromatic compounds such as furanols in various organisms. Furanone compounds play a crucial role in regulating fruit aroma (Perez-Roman et al., 2022), while the 2-Methylenefuran-3-one reductase regulatory gene has been demonstrated to play a key role in plant responses to disease stress (Rego et al., 2022). This gene was found to co-express with proteins associated with the cell wall biosynthesis pathway during plant disease stress response (Perdiguero et al., 2017). This co-expression may promote plant height and leaf width growth, thereby indirectly influencing photosynthetic rates and sustaining plant growth under LK stress. Furthermore, robust photosynthetic performance promotes increased synthesis of organic acids and osmotic regulators in plants, enhancing K redistribution capacity within the plant body (Panchal et al., 2021). The stable expression of these proteins helps maintain the integrity of photosystem centers, ensuring efficient light energy conversion and thereby sustaining net photosynthetic rates. And collectively provide intermediates for plant growth, jointly supporting cell division and expansion, and alleviating leaf narrowing and reduced leaf area caused by LK levels. And they indirectly influence overall K use efficiency in plants by maintaining ATP supply and membrane potential stability within leaves.

In the shoot MEturquoise module, we also selected *NIASHv1049C03*, *LOC123425161*, *LOC123448799* and *NIASHv3101N17*—the four genes most strongly correlated with H^+^, K^+^-ATPase activity (r = 0.91). The proteins predicted for these genes in NCBI are respectively: probable U3 small nucleolar RNA-associated protein 7 isoform X1 (*NIASHv1049C03*), DEAD-box ATP-dependent RNA helicase 36 (*LOC123425161*), probable pre-mRNA-splicing factor ATP-dependent RNA helicase DEAH3 isoform X1 (*LOC123448799*), probable pre-mRNA-splicing factor ATP-dependent RNA helicase DEAH4 (*NIASHv3101N17*). Similarly, these predicted genes have not been extensively studied, but there are relevant reports on the proteins they predict. The U3 nucleolar RNA participates in the maturation process of the small subunit rRNA through designed base pairing with pre-rRNA (Hughes, 1996). Under LK stress conditions, cellular energy is prioritized for ion transport and stress response systems. Simultaneously, multiple RNA-binding proteins such as U3 small nucleolar RNA-associated protein regulate various developmental processes by modulating the expression of specific transcripts, thereby enabling rapid control over H^+^,K^+^-ATPase expression (Lorković, 2009). DEAD helicases constitute the largest family of RNA helicases within the helicase superfamily 2. In plants, they primarily facilitate RNA structural transitions and participate in RNA remodeling and the reconfiguration of RNA-protein complexes (He et al., 2024). DEAD helicase and its corresponding ATPase perform multiple cellular functions in RNA metabolism and act as metabolic sensors to catalyze a wide range of cellular functions (Putnam and Jankowsky, 2013). ATP-dependent RNA helicase 36 significantly participates in ribosomal biogenesis regulation under LK stress by reshaping RNA secondary structures to promote proper folding and splicing of rRNA precursors (Nawaz and Kang, 2017). The ATP-dependent RNA helicase DEAH3/DEAH4, a probable pre-mRNA splicing factor, regulates pre-mRNA splicing efficiency through its ATP-dependent hydrolase activity. It plays a crucial role in the expression of stress-responsive genes, such as Ca^2+^ channel proteins and CBL-CIPK signaling components, by generating specific splicing isoforms, regulating calcium signaling pathway dynamics, thereby enhancing plant stress responses (Lee et al., 2024). Three genes encoding ATP-dependent RNA helicase proteins may regulate the expression efficiency of precursor RNA transcription and sense K stress signals to ensure H^+^-K^+^-ATPase expression. This promotes plant uptake of external K and facilitates internal K redistribution in response to external K deficiency. Shoot cells maintain ion homeostasis, energy metabolism, and protein synthesis homeostasis by regulating H^+^,K^+^-ATPase enzyme activity in concert with multiple functional proteins (Tang et al., 2021). In summary, under LK stress, barley shoot tissues enhance H^+^,K^+^-ATPase-driven transmembrane transport capacity and promote K redistribution by coordinating the expression of RNA processing, ribosome biogenesis, and ion pump proteins expression. Concurrently, they regulate calcium signaling and energy utilization efficiency through RNA helicase-mediated signaling networks, thereby achieving multi-level adaptation from gene expression to physiological function.

AS is the process by which two or more distinct transcripts are generated from the same precursor mRNA molecule through the use of different splicing sites (Mastrangelo et al., 2012). Through this mechanism, a single gene can produce multiple transcripts and protein isoforms, playing a crucial role in gene expression regulation (Zhang R. X. et al., 2017). Some studies indicate that AS extensively induces genes involved in both biotic and abiotic stress responses to enhance plant stress tolerance (Cui et al., 2017). And the AS event in plants primarily focuses on abiotic stresses such as drought or heat stress (Rosenkranz et al., 2022), salt stress (Yu et al., 2021) and metabolic regulation in plants (Lam et al., 2022). A study on cassava revealed that under salt stress conditions, the transcription of serine/arginine-rich (SR) protein genes exhibited elevated expression, accompanied by significantly increased alternative splicing. This suggests that SR proteins may be involved in the plant’s adaptation to salt stress (Gu et al., 2020). Additionally, A study in *Arabidopsis thaliana* revealed that LK stress induced an increase in the abundance of the MYB59α subtype, while the overall transcript abundance remained unchanged. This indicates that MYB59α responds to LK stress by altering its splicing pattern (Enomoto et al., 2023). In this study, four of these 12 hub genes are directly or indirectly involved in the process of alternative splicing. Therefore, we systematically organized these 12 gene alternative splicing events and found that only three genes—*LOC123407914* (peptidyl-prolyl cis-trans isomerase, chloroplastic isoform X1), *NIASHv1113H14* (glycerate dehydrogenase) and *LOC123448799* (probable pre-mRNA-splicing factor ATP-dependent RNA helicase DEAH3 isoform X1) exhibited differential alternative splicing with upregulation or downregulation in their shoot parts. Based on these three genes, we expanded our analysis from the transcriptome sequencing results to include 12 genes associated with K^+^ absorption or transport, all of which are candidates for alternative splicing (**Supplementary Figure S6**). These primarily include calmodulin, potassium-sensitive proteins, zinc-induced promoter-like families and ADP-ATPase proteins. The CIPK protein family has been shown to activate HA5K proteins during K deficiency in plants to enhance K^+^ uptake (Ragel et al., 2015). Furthermore, the AKT1L protein, which interacts with CIPK, plays a crucial role in promoting K^+^ absorption and strengthening plant stress resistance (Lin et al., 2021). In addition, CIPK family proteins, which interact with CBL, have been extensively studied in plant responses to abiotic stress, calcium-regulated carbon and nitrogen metabolism and signal transduction (Grzechowiak et al., 2020; Das et al., 2024; Narwal et al., 2024). Early studies demonstrated that ZIFL maintains gene expression patterns in rice and plays a crucial role in zinc and iron homeostasis (Ricachenevsky et al., 2011). Recent research indicates it also exerts significant influence on the development of crop traits (Meena et al., 2021). CBSX2 is a regulator of thiol oxidoreductase (Murai et al., 2021), playing a crucial role in modulating target protein activity in response to light signals. The activity of chloroplast ATP synthase (CFoCF1) is regulated by the redox state of its gamma subunit (Li et al., 2023), and its alternative splicing may be subject to feedback regulation by the *FLbaf146l04* gene (chloroplast ATP synthase subunit gamma). The results indicate that these 12 genes and their potentially regulated associated genes or proteins may adapt to environmental stress through alternative splicing under hypokalemic stress. However, significant gaps remain in research on these genes and the alternative splicing they may regulate. Many mysteries persist regarding the mechanisms by which genes capable of undergoing alternative splicing events regulate K^+^ uptake in plants under K stress. Further research on these genes may elucidate the molecular genetic mechanisms by which plants respond to LK stress through alternative splicing.

Among the 12 bub genes identified across three modules in this study, the four genes in MEbrown primarily play crucial roles in stress responses and signaling molecule transmission. Represented by the gene *NIASHv2003M06*, which is predicted to encode the SDE2 protein, these genes enable plants to maintain the activity of root apical meristem tissues under LK stress. This ensures the sustained expression of proton pump genes, thereby preserving the plant’s ability to absorb K^+^. The four MEpink genes primarily function in cell walls and chloroplasts—organelles associated with photosynthesis—while maintaining cellular redox states and energy levels under stress conditions. Among them, the gene *FLbaf146l04*, predicted to encode the chloroplast ATP synthase subunit gamma, directly participates in plant photosynthesis. Its expression pattern may reflect a plant strategy to regulate photosynthetic efficiency, maintain leaf width, and ensure energy synthesis capacity in response to K deficiency. The four genes of MEturquoise primarily function at the levels of RNA helicase and the nucleolus within the cell nucleus. They serve as ATP-dependent precursor RNA splicing factors, promoting the correct splicing of rRNA precursors by reshaping RNA secondary structures. U3 nucleolar RNA participates in the maturation of the small subunit rRNA through specific base pairing with precursor rRNA. The coordinated action of these four genes ensures normal protein expression under stress conditions, thereby maintaining the proper functioning of plant cells. Further research on these genes may provide new insights into how plants cope with LK stress, potentially through signal transduction, photosynthesis-related organelles, and nuclear protein expression.

## 5 Conclusion

This study investigated the effects of LK stress on the growth of three barley varieties from phenotypic, physiological, and transcriptomic perspectives. The results indicated that LK stress reduced K accumulation, plant height, root surface area, dry weight, and photosynthetic parameters in all three barley varieties, thereby inhibiting barley growth. Significantly enhanced shoot K transfer coefficient and H^+^,K^+^-ATPase activities, indicating their crucial roles in alleviating LK stress in barley. Transcriptome analysis indicates that LK stress primarily affects hormone signal synthesis and transduction, antioxidant enzymes, and transcription factors. Differentially expressed genes are mainly involved in plant defense and immunity, metabolite and energy regulation, photosynthesis, carbohydrate and nitrogen metabolism, as well as hormone and developmental regulation. The 12 hub genes identified by WGCNA analysis maintain redox homeostasis and amino acid metabolism within plant cells by acting on H^+^, K^+^-ATPase and its upstream signaling molecules, photosynthesis-related pathways, and stress responses, thereby enhancing K use efficiency. Further research on these pivotal genes may provide new insights into signal transduction, photosynthesis-related organelles and nuclear protein expression regulation during plant responses to K deficiency.

## Data Availability

The original contributions presented in the study are publicly available. This data can be found here: https://www.ncbi.nlm.nih.gov/sra, accession number PRJNA1431199.

## Supplementary material

Supplementary Table 1 Sequencing output statistics table.

Supplementary Table 2 Sequence alignment efficiency statistics table.

Supplementary Table 3 Sequencing data filtering statistics table.

Supplementary Table 4 Statistical analysis of regional distribution table.

Supplementary Table 5 Gene FPKM Statistics table.

Supplementary Table 6 GO information table for DEGs in shoots and roots.

Supplementary Table 7 KEGG information table for DEGs in shoots and roots.

Supplementary Table 8 GO information table for GSEA in shoots and roots.

Supplementary Table 9 KEGG information table for GSEA in shoots and roots.

Supplementary Table 10 Statistical table of genes highly associated with corresponding phenotypes across three modules.

Supplementary Table 11 Root MEbrown hub gene screening table.

Supplementary Table 12 Shoot MEpink hub gene screening table.

Supplementary Table 13 Shoot MEturquoise hub gene screening table.

Supplementary Table 14 Table of qPCR primers for 12 genes and the Hvactin gene.
