# Supplementary material for: Transcriptome and weighted gene co-enrichment analysis revealed modules and candidate genes associated with barley response to low potassium stress
Source: Front Plant Sci. 2026 Mar 17;17:1779943. doi: 10.3389/fpls.2026.1779943 (PMC13035491; doi:10.3389/fpls.2026.1779943)
Supplement: Supplementary file 1 [file Supplementaryfile1.docx]

Supplementary Material
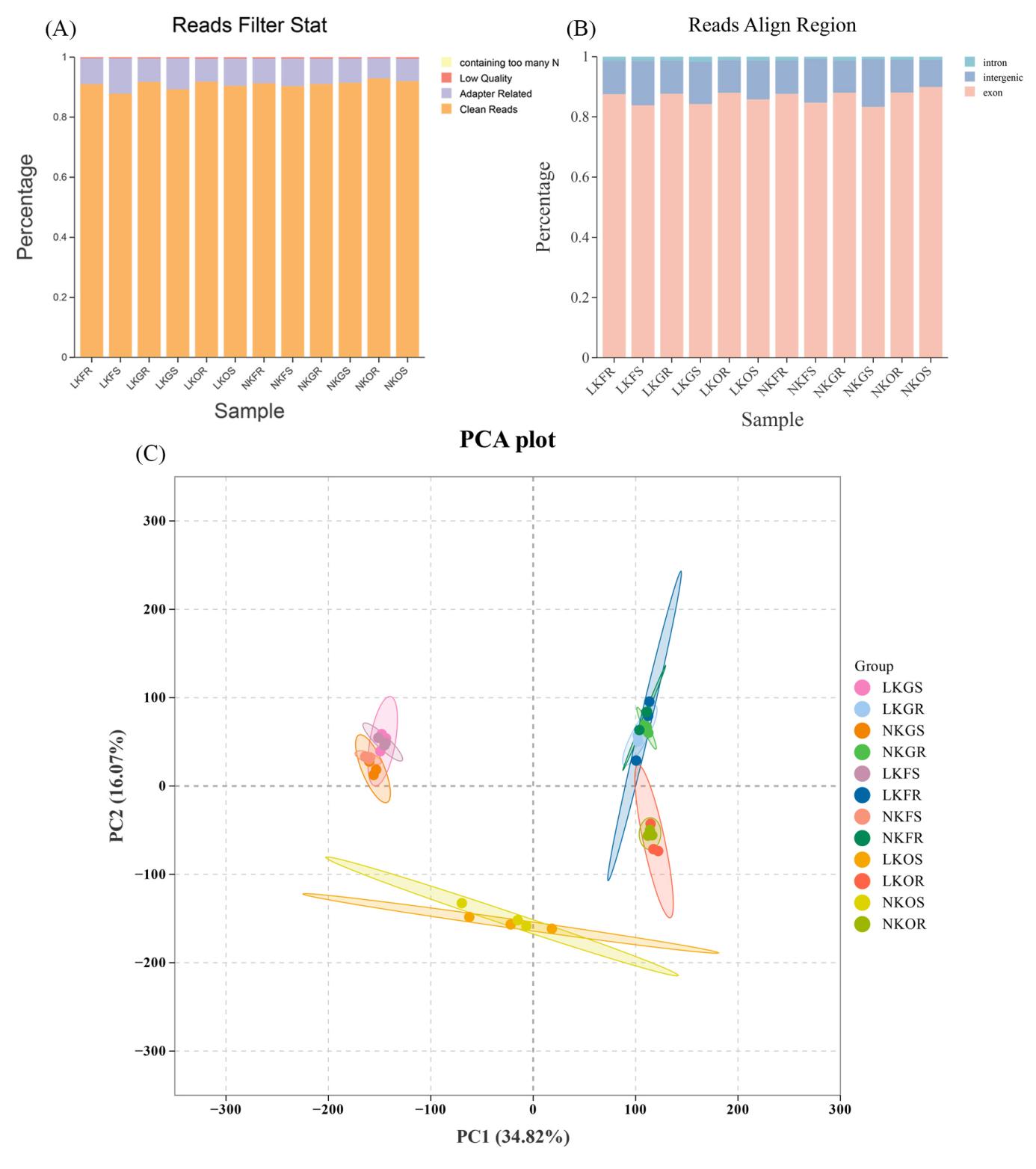


**Supplementary Figure 1.** RNA-Sequencing data quality map. (A) Read filter stat in each sample. (B) The coverage of genome alignment and gene location. (C) Relationship between sames.


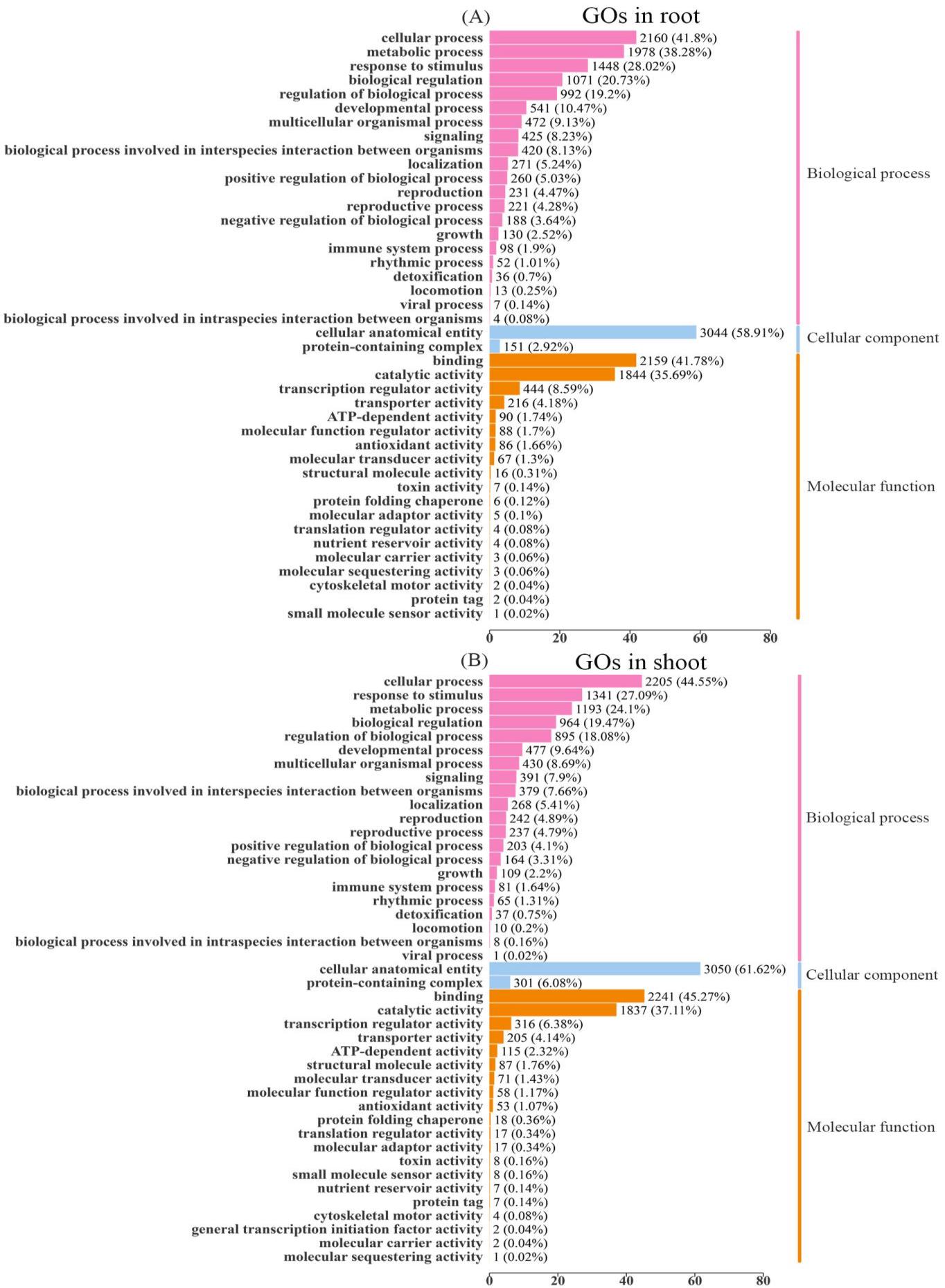


**Supplementary Figure 2.** GO enrichment analysis plots for different sample group. (A) GO enrichment analysis of DEGs of root in Franklin, Grimmet and CN0126. (B) GO enrichment analysis of DEGs of shoot in Franklin, Grimmet and CN0126.

**
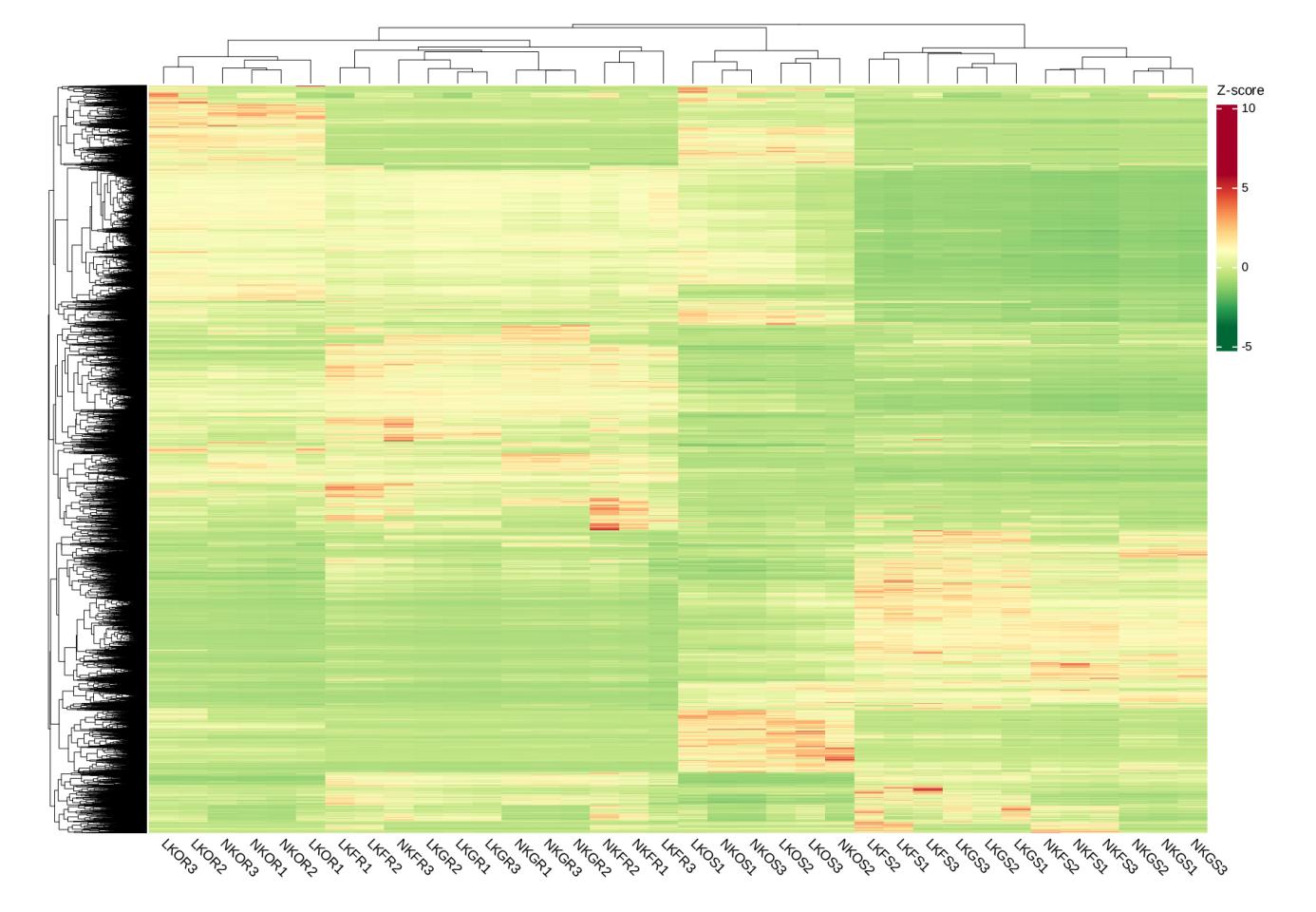
**

**Supplementary Figure 3.** Clustering Heatmap of All Sample DEGs.


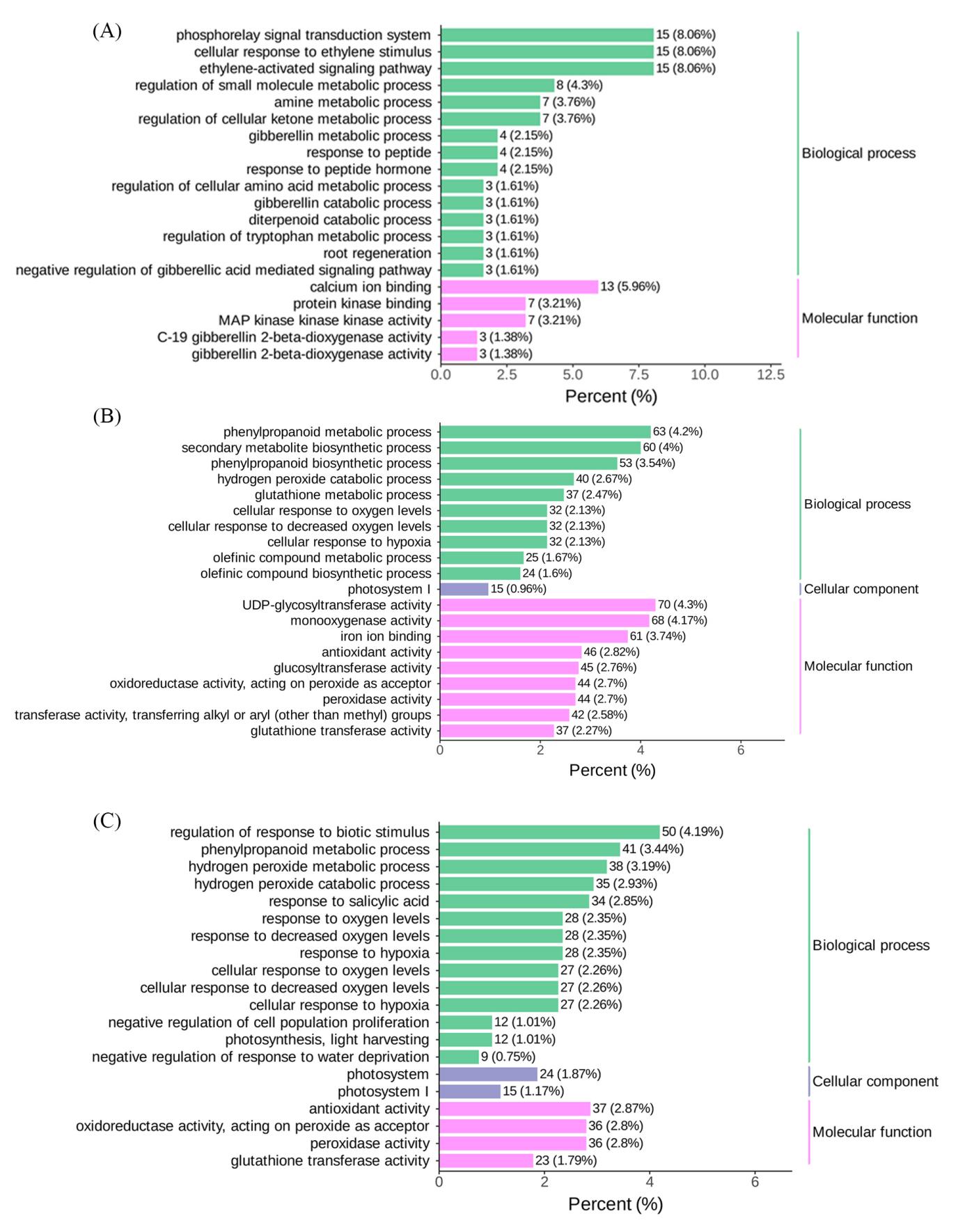


**Supplementary Figure 4.** GO enrichment analysis plots for each sample group. (A) GO enrichment analysis of DEGs of root in Franklin. (B) GO enrichment analysis of DEGs of root in Grimmet. (C) GO enrichment analysis of DEGs of root in CN0126.


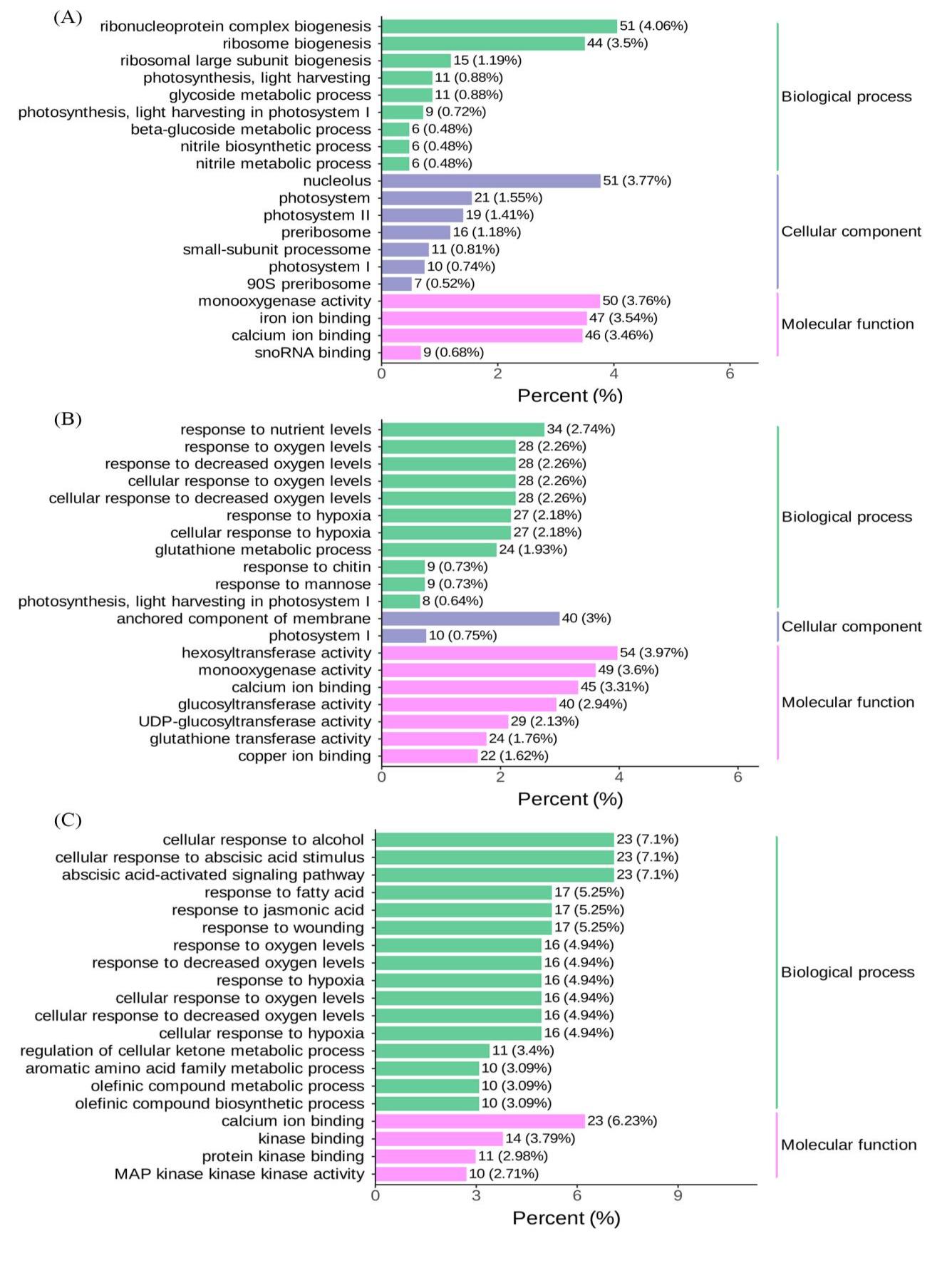


**Supplementary Figure 5.** GO enrichment analysis plots for each sample group. (A) GO enrichment analysis of DEGs of shoot in Franklin. (B) GO enrichment analysis of DEGs of shoot in Grimmet. (C) GO enrichment analysis of DEGs of shoot in CN0126.


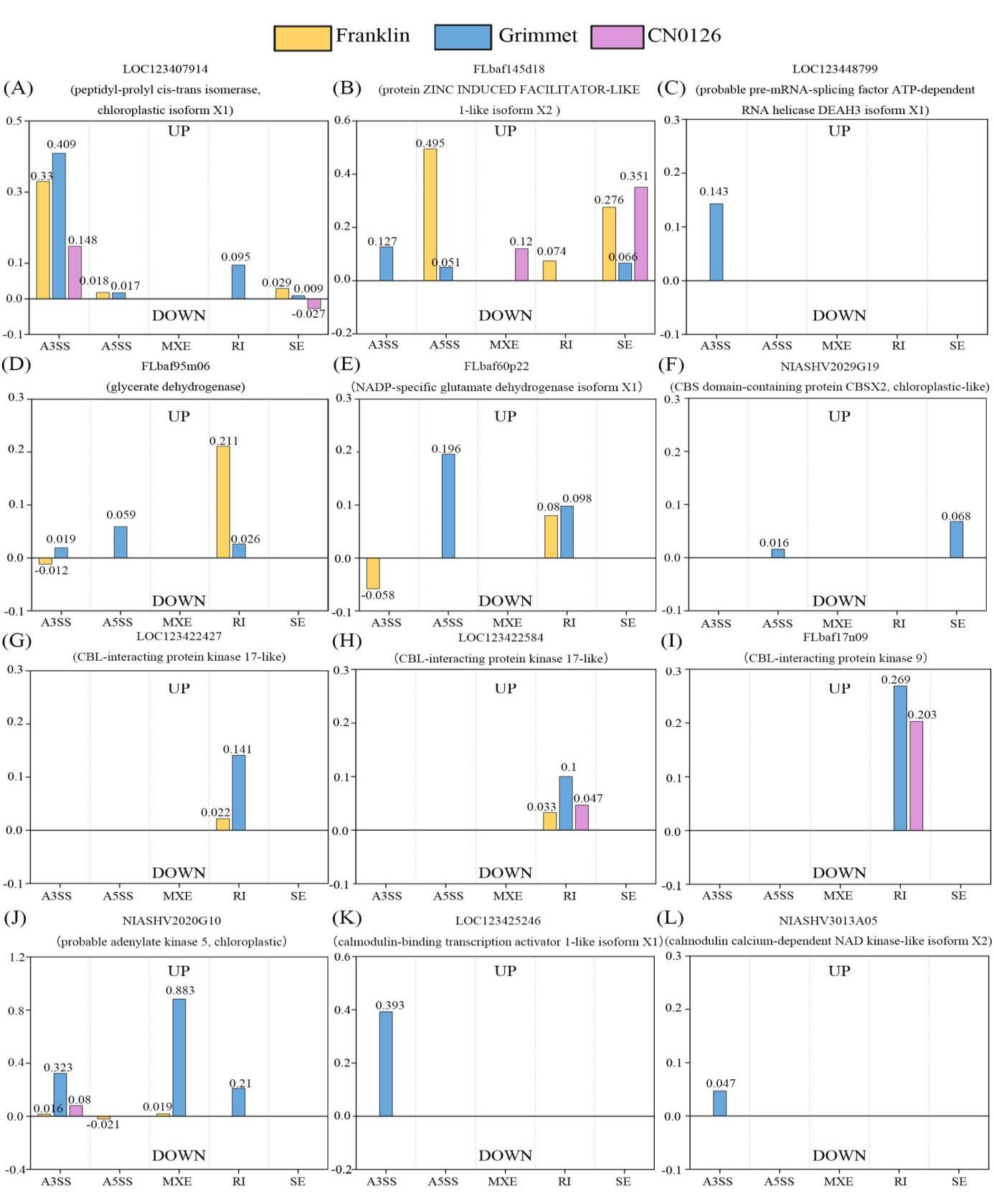


**Supplementary Figure 6.** Upregulation and downregulation of Alternative Splicing events in selected genes across three varieties. The criterion for determining upregulation or downregulation is as follows: subtract the level of the corresponding variable shear event in the control group from the level of the variable shear event in the treatment group. If the result is greater than 0, it is considered upregulation, otherwise, it is downregulation.
